# Zeatin: The 60th anniversary of its identification

**DOI:** 10.1093/plphys/kiad094

**Published:** 2023-02-15

**Authors:** Paula Elizabeth Jameson

**Affiliations:** School of Biological Sciences, University of Canterbury, Private Bag 4800, Christchurch 8140, New Zealand

## Abstract

While various labs had shown cell division-inducing activity in a variety of plant extracts for over a decade, the identification of zeatin (Z) in 1964, the first known naturally occurring cytokinin, belongs to Letham and co-workers. Using extracts from maize (*Zea mays*), they were the first to obtain crystals of pure Z and in sufficient quantity for structural determination by MS, NMR, chromatography, and mixed melting-point analysis. This group also crystallized Z-9-riboside (ZR) from coconut (*Cocos nucifera*) milk. However, their chemical contributions go well beyond the identification of Z and ZR and include two unambiguous syntheses of *trans*-Z (to establish stereochemistry), the synthesis of ^3^H-cytokinins that facilitated metabolic studies, and the synthesis of deuterated internal standards for accurate mass spectral quantification. Letham and associates also unequivocally identified Z nucleotide, the 7-and 9-glucoside conjugates of Z, and the *O*-glucosides of Z, ZR, dihydro Z (DHZ) and DHZR as endogenous compounds and as metabolites of exogenous Z. Their contributions to the role of cytokinins in plant physiology and development were also substantial, especially the role of cytokinins moving in the xylem. These biological advances are described and briefly related to the genetic/molecular biological contributions of others that established that plants have an absolute requirement for cytokinin.

## Teaser

D. Stuart Letham and co-workers identified zeatin in 1964 and in the following decades revealed the complexity of the cytokinins in both their biochemistry and their effects on growth and development.

## Introduction

**Figure 1.**
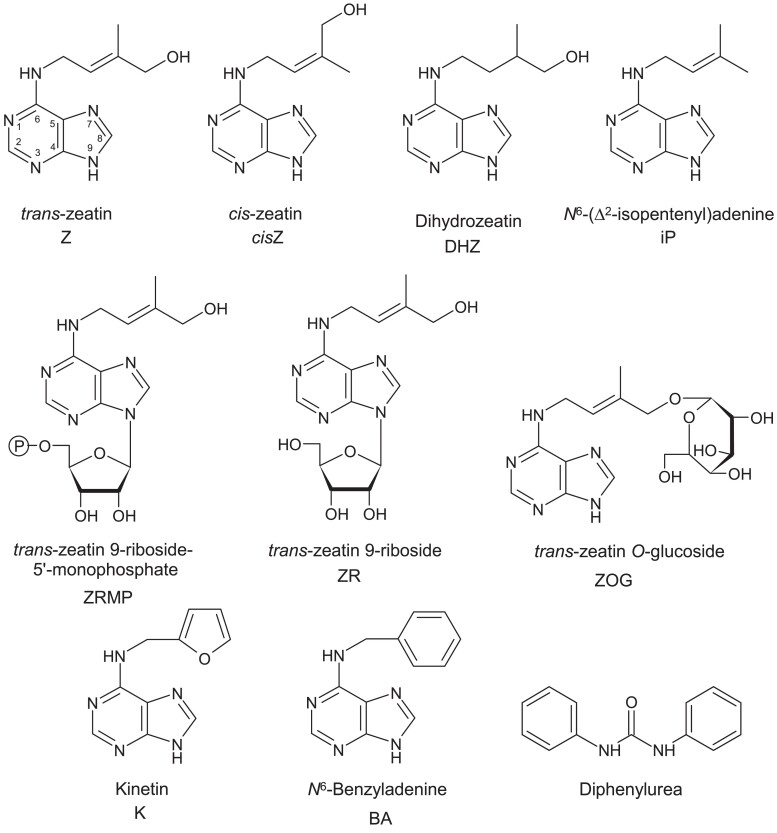
Chemical structures of some naturally occurring cytokinins, kinetin (K), and *N*,*N*′-diphenyl urea (DPU). The free bases, Z, *cis-Z*, DHZ, and iP, are regarded as the biologically active forms. These forms all exist as ribosides and nucleotides. The numbering of the purine ring is shown for zeatin and shows the point of attachment for the 7- and 9-glucosides. Tomáš Werner kindly provided the structures.

This article outlines the pioneering work that culminated in the identification of zeatin (Z) (Fig. 1) by David Stuart Letham and co-workers (Letham et al. 1964), the first cytokinin identified in extracts from plants. The article includes accounts from Letham and represents a wish by the author to highlight the substantial contributions that Letham and co-workers have made to cytokinin chemistry and biology and to counteract both the verbal assertions by Folke Skoog that Letham “stole Miller's data” and the written assertion by Skoog that Carlos Miller should be credited with the “isolation and composition” of Z (Skoog 1994), noting that these were statements with which Miller himself disagreed (R.M. Amasino pers. comm., 2023). The reader will see how one person (Letham) with little support and limited facilities opened an impasse for exploitation by the instrument-based brilliance of others (Shannon and McDonald). This is the story of the discovery of Z as a chemical entity and early discoveries that pointed to the critical role of cytokinins in plant physiology and development.

There are also some take-home messages to those embarking on cytokinin investigations today who are under the misapprehension that quick analyses of inadequately extracted, un- or poorly purified and un- or poorly separated samples can give an accurate picture of the cytokinin content of a sample.

## The role of cell division and cell size in the postharvest storage of apple (*Malus domestica*) fruit

Letham became associated with cell division research in a very indirect way. His position at the Mt Albert site of the Department of Scientific and Industrial Research (DSIR), Fruit Research Division (FRD), in Auckland, New Zealand, began in 1955 and involved investigating cold storage disorders of apple fruit. The work at Mt Albert was done in a temporary laboratory termed “the barn” built as an additional floor over a storage shed for farm machinery (see Ferguson and Bellamy 2011). Letham investigated the effect of fertilizer treatment on fruit composition and physiology, in relation to the incidence of internal breakdown during cold storage (Letham 1961, 1969a). Of 20 fruit attributes, reduced cell size, elevated P content, and reduced respiration per cell were the only fruit attributes associated with a low incidence of internal breakdown. Letham had earlier devised methods for the maceration of tissue composed of easily damaged cells and to achieve this without change in cell volume. The macerating action of sodium ethylene-diamine-tetraacetate was attributed to the ability of the reagent to dissolve the insoluble salts of pectic and low-methoxyl pectinic acids that appeared to form a large part of the middle lamella (Letham 1958a, 1960a). This technique provided suspensions for cell counting and was used to show that cell volume was positively correlated with breakdown incidence (Letham 1961). Phosphorus fertilizer increased *P* content of fruit, while *N* fertilization reduced the *P* content. Elevated *P* content of fruit was associated with reduced cell size and reduced breakdown during cold storage (Letham 1969a).

As it was clearly desirable to promote cell division, Letham investigated the promotion of cell division in in vitro culture. Letham (1958b, 1960b) published the first successful in vitro cultivation of six pome-fruit tissues. Apple fruit tissue, sampled either before or after the cessation of cell division, grew as undifferentiated tissue in culture but only in the presence of a cell division-inducing factor, 2,4-D and other nutrients. Growth was stimulated by the addition of coconut (*Cocos nucifera*) milk, plant extracts (especially that from immature maize (*Zea mays*) seed), or 6-furfurylaminopurine (kinetin) (Letham 1958b). There was interest in the identification of the natural stimulants of cell division, which appeared more effective than kinetin, in the apple tissue cultures.

By the mid-1950s two compounds that stimulated cell division had been identified: kinetin and *N*,*N*′-diphenyl urea (DPU) (Fig. 1). This latter compound was claimed to occur in coconut milk but is now attributed to contamination (refer to later section on coconut milk). Carlos Miller, working in Folke Skoog's lab, had isolated and identified kinetin from autoclaved herring sperm DNA (Miller et al. 1955a, 1955b, 1956; see review by Amasino 2005). Kinetin is a strong cell division-inducing factor, and its identification resulted in the development of purely synthetic media that included auxin. Depending on the ratio of the auxin and kinetin, shoot or root organogenesis or undifferentiated growth resulted (Skoog and Miller 1957). Kinetin forms in all cells by very slow natural degradation of DNA (Barciszewski et al. 1997). There is no evidence that this kinetin has a regulatory function, nor is it found ubiquitously in plant tissues (Strnad 2021).

Letham (a chemist by training) was interested in identifying the cell division factor that was clearly present in a number of plant species, including fruit, seeds, and coconut milk, as detected by biological assays. This interest was in spite of comments such as “If numerous major overseas laboratories are purifying this unknown hormone, why waste time trying to do it in New Zealand with very limited facilities?” Table 1 lists nine groups and the tissues they were focusing on. However, Letham was encouraged by Ted Bollard (his supervisor at DSIR) but told, initially, that he could not work on coconut milk as that was F.C. Steward's area, as well others, including Van Overbeek in California (Table 1 and see citations in Steward and Shantz 1959). Even though DSIR was working on storage disorders of apples, apples were also out, as in Canberra, CSIRO had a large group working with these (Table 1; Goldacre and Bottomley 1959).

**Table 1.** Key personnel and laboratories actively purifying natural cytokinins around 1960

Laboratories actively purifying natural cytokinins about 1960—when DSIR entered the field.		
Principal workers	Location	Tissue/fluid extracted
E.M. Shantz, F.C. Steward	Cornell, USA	Coconut milk; sweet corn; *Aesculus* immature fruit
J.E. Kloeffler, J. van Overbeek	Shell Research	Coconut milk
	Modesto, USA	
A. Kovoor	Paris, France	Coconut milk
H.N. Wood	New York, USA	Crown gall tumor tissue
E. Maia	Versailles, France	Tomato fruit
G. Beauchesne, R. Goutarel	Angers, France	Immature maize
N.P. Kefford, J.A. Zwar	CSIRO	Apple fruitlets; coconut milk
	Canberra, Australia	
J.H. Rogozinska, J.P. Helgeson, F. Skoog, J.A. Zwar	Wisconsin, USA	Pea blanch water from 3,000 kg fresh peas; geminating peas
C.O. Miller, F.H. Witham	Indiana, USA	Immature maize seed

An early attempt to purify cytokinins from coconut meal was made by Skoog and associates in the early 1950s (Mauney et al. 1952). In 1953, Miller attempted purification from yeast extract (see Amasino 2005).

As storage disorders in apples appeared to be associated with cell number, Letham had been working with apples across the cell division period. Letham and Bollard (1961) detected a compound that resembled kinetin as it interacted synergistically with IAA in their bioassay using explants from secondary phloem of carrot (*Daucus carota* subsp. *sativus*) roots. Letham (1963a) showed that the activity increased during the cell division period of apples and declined markedly about the time of cessation of division. He suggested that a reduction in cell division-inducing activity may be at least partly responsible for the cessation of division in the apple fruitlet. Developing plum (*Prunus* sp.) fruitlets exhibited a similar relationship between cell division in the fruitlets and extracted cytokinin activity. The cell division-inducing factor from plums was distinguished from kinetin both chemically and biologically, and strongly increased the cell number in carrot explants (Letham 1963b). It is noteworthy that in this paper Letham suggested the term “cytokinin” be adopted for those compounds exhibiting kinetin-like activity, a term subsequently adopted (Skoog et al. 1965).

## Purification and identification of cell division factors

Today, high-pressure/performance liquid chromatography (HPLC) and thin-layer chromatography are two powerful techniques routinely used for purification of organic compounds, but in 1960, when Letham initiated purification of natural cytokinins, these methods were not available. His aim to obtain natural cytokinins (trace substances) in pure crystalline form (crystallized to constant melting point) appeared to have little prospect of success. This prospect is supported by the lack of success by the nine labs listed in Table 1 which were attempting to purify cytokinin.

**Figure 2.**
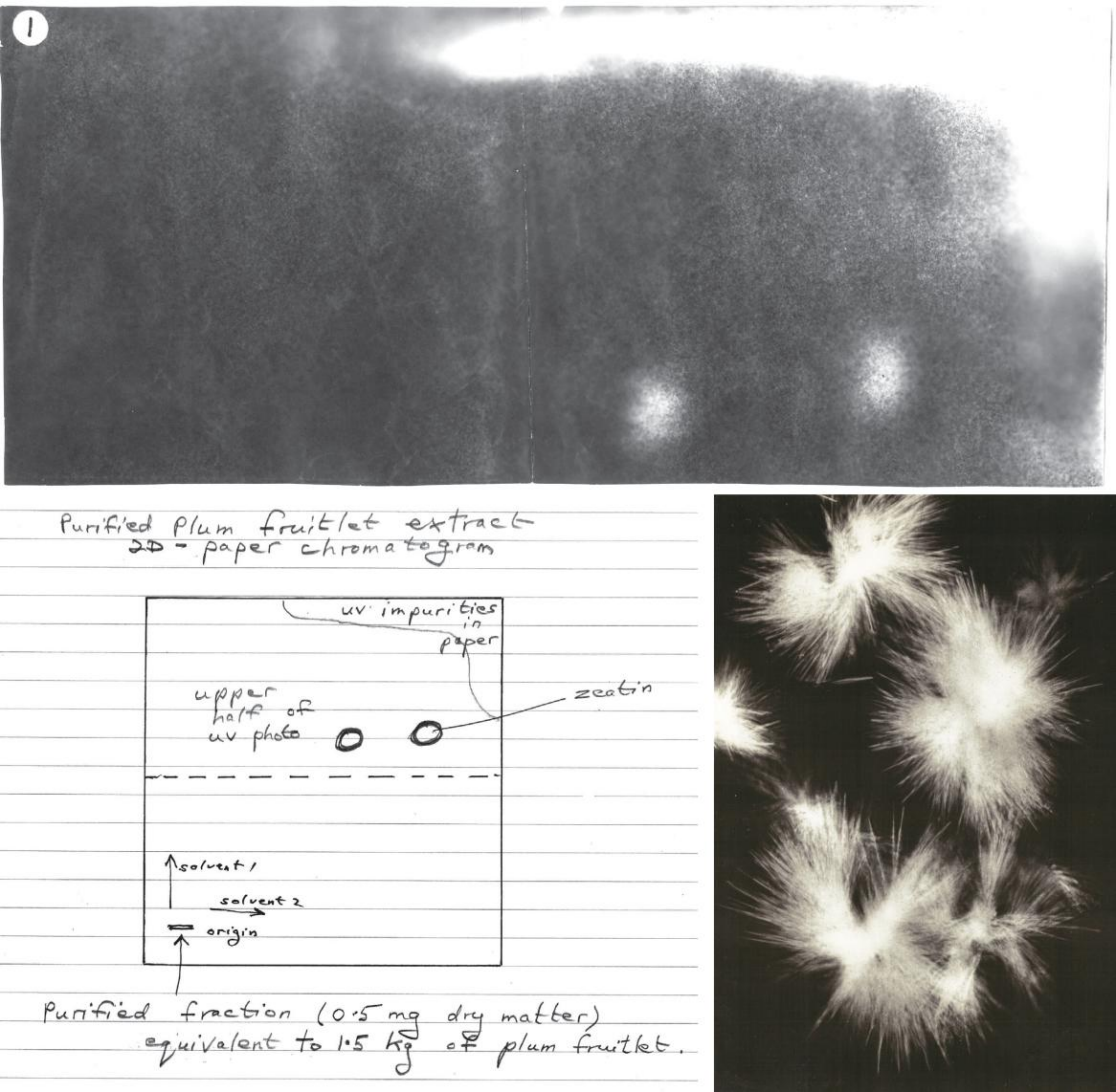
UV-absorbing compounds in purified plum extract and crystals of zeatin picrate from *Z. mays*. Purified plum extract (0.5 mg dry matter, equivalent to 1.5 kg plum fruitlets) was applied near the corner of a 40 × 40 cm sheet of Whatman No.1 filter paper. The chromatogram was developed in two dimensions with different solvents. After drying, the filter paper was placed over a sheet of photographic paper and exposed to filtered UV light for several minutes. When developed, two UV-absorbing spots were revealed as white areas in the upper half of the photographic paper. Letham states “the UV spot on the right was a purine (about 5 μg) and at only 0.1 μg/L of culture medium induced marked cell division in a cytokinin bioassay”. Top: The upper half of the original exposed photographic paper. Lower left: Hand-drawn image of the filter paper by Letham. Lower right: Photomicrograph of the crystals of the picrate of zeatin from *Z. mays*. “The key to the purification and identification of zeatin lay in the crystallization step to form the picrate of zeatin” (Letham 1963c; 1964).

The activity derived from plums appeared to be due to one unknown cytokinin which was purified by Letham as outlined here. The fractionation of extracts was guided by cytokinin bioassays with carrot phloem tissue cultures. An aqueous solution of plum fruitlet ethanol extract was first further extracted with ethyl acetate to remove strong inhibitory activity leaving a highly active aqueous phase (see Letham 1963b, 1964). Sequential purification by the following steps then followed: chromatography using a cation ion exchange resin (H^+^ form), precipitation with silver ions, cellulose column chromatography, and extensive two-dimensional paper chromatography on numerous large, thick, washed sheets. This yielded a UV-absorbing compound (termed A1) (Fig. 2) with a UV spectrum characteristic of N^6^-substituted adenines. A1 interacted with *myo*-inositol and auxin to induce cell division. The amount obtained of this compound was estimated to be about 0.2 mg per 30 kg of plum fruitlets. While *myo*-inositol was separately purified and crystallized from the plum extracts (Letham 1964, 1966a), there was an insufficient quantity of Factor A1 for identification by mass spectrometry (MS), so Letham turned to *Z. mays*.

### Cell division factor in *Z. mays*

Immature grains from *Z. mays* had been identified as a source of plant growth substances (e.g. Letham 1958b; reviewed in Steward and Shantz 1959) and, based on Miller's (1961) results, *Z. mays* had a much higher level of cytokinin than fruit tissues.

Miller (1961) reported the partial purification of an active fraction from *Z. mays*—a UV-absorbing band showed activity in the soybean (*Glycine max*) callus bioassay. UV absorption data indicated that the fraction contained a substituted adenine, but in his 1961 paper, Miller noted that crystals were not obtained. Further details of their progress were provided by Miller and Witham at the 1963 Régulateurs de la Croissance Vegétalé conference in Gif-sur-Yvette, France. The text of their contribution is presented in Supplemental File S1A. This paper was omitted from the published volume and was published subsequently as an Addendum. They estimated to have obtained 0.03 to 0.1 mg of factor per kg of milky-stage *Z. mays* kernels. Following various chemical treatments, they concluded that the factor was probably a 6-(substituted) aminopurine. Miller and Witham (1964) made suggestions as to the nature of the substituent which Letham provided comment on (see annotated Supplemental File S1B). Miller and Witham (1964) stated that the second carbon of the substituent was subject to attack by KMnO_4_ and suggested this may mean a double bond between the second and third carbon. Other reactions led to additional suggestions being made by Miller and Witham that the factor may possess an OH group or another group that can give rise to it, and the nuclear magnetic resonance (NMR) spectrum included a peak very likely attributable to a group in which an oxygen atom is attached to a carbon atom.

Letham writes:

The nmr spectrum of zeatin is now well defined. The most prominent peak is a singlet due to the methyl group. Miller and Witham (1964) did not detect this, indicating their cytokinin sample was grossly impure, an observation confirmed by the abnormally high UV absorbance at low wavelengths observed in their spectra. Unfortunately, they had reached an impasse in their purification, just as Skoog and co-workers did in their purification of pea seed cytokinin (Rogozinska et al. 1965). To progress further in 1963, they perhaps required a very specific precipitation and recrystallisation step. One based on picric acid is described below.

As discussed below, the key to obtaining the complete structure was a mass spectroscopic analysis of crystalline zeatin. Miller and Letham were working in parallel to obtain crystals (R.M. Amasino, pers comm., 2023), and Letham was the first to successfully crystallize zeatin and obtain definitive mass spectra (Letham et al. 1964).

Miller and Witham (1964) considered the possibility that the compound being studied might be made during the isolation process and also stated that the compound represented only a small fraction of the total activity in the original extract. They also suggested that if it is truly naturally occurring then derivatives such as nucleosides, nucleotides, or even more complicated ones probably exist. Bioassay data indicated more of the factor was present in kernels than in vegetative tissues. Young sunflower (*Helianthus annuus*) fruits and a soybean protein enzymatic hydrolysate had identical properties. Miller and Witham (1964) concluded: “Probably either the compound or material from which it is made is widespread in plants.”

Letham (1963c), who had previously used extract from immature sweet corn (a *Z. mays* variety) seeds as a cell division-inducing activity in apple tissue cultures, now turned to the purification of cytokinin from sweet corn. Sweet corn was more readily available and, since it lacked the growth inhibitors of plums, the extraction procedure for the two sources differed considerably. The sweet corn kernels were excised using a sweet corn stripping machine (borrowed from a local cannery) and frozen. Samples of 60 to 70 kg were extracted with 200 L of purified ethanol. All fractionation of extracts was followed by carrot phloem bioassay. The clarified extract was applied directly to a large column of cation exchanger resin (H^+^ form) and the concentrated column eluate was subjected to precipitation with Ag^+^. The recovered fraction was subjected to a simplified procedure involving two one-dimensional paper chromatography purifications using eight or nine large sheets of thick paper for both steps.

A key feature was the prewashing of paper chromatograms to remove impurities, but the most critical advance was the final step involving the precipitation of the active factor (M) as a picrate. Recrystallization of the picrate yielded clusters of fine needles (Fig. 2). The yield was 4.2 mg from 70 kg of sweet corn. An impasse in purification had been resolved. The melting point and spectral characteristics of the picrate of M were provided (Letham 1963c, 1966b). The hydrochloride and free base of M were prepared from the crystalline picrate. All three compounds induced cell division in carrot secondary phloem explants in the presence of IAA and *myo*-inositol with very similar activity.

A comparison of the crystalline free base with kinetin in the bioassay showed that, while kinetin had slight activity at 1 μg/L of the medium, the isolated base increased cell number about 6-fold and showed activity even at 0.01 μg/L (Letham 1966a). Letham proposed the name zeatin (Z)—not because it was isolated in crystal form in New Zealand but because it was isolated from *Zea mays* (1963c). He commented that, while the relationship of Z to Miller's partially purified factor seemed uncertain, he thought it likely they were identical. He also commented that, as Z and his plum factor (A1) appeared to be identical, Z may be a growth factor of wide occurrence (Letham 1963c; submitted June 1963).

After purification of Z picrate in 1963, it was Letham's good fortune to be invited to the 5th International Conference on Plant Growth Regulators (Gif-sur-Yvette, France, July 15–20 1963). His conference contribution (Letham 1964) is presented in Supplemental File S2 and discusses the plum and *Z. mays* factors. Notably Figure 5 in this paper shows a photomicrograph of the crystals of the picrate of Z from sweet corn, photos of the carrot secondary phloem bioassay of Factor M (zeatin) from sweet corn, and Table 1 provides the cell number per explant showing the strong synergism between factor M and the basal medium containing IAA and *myo*-inositol. 1,3-diphenylurea (2 mg/L) was not active in the bioassay, nor was kinetin at 1 μg/L of the medium, whereas Z at the same concentration induced large increments in both weight and cell number. Letham (1964) concluded from a variety of chemical tests that it was unlikely that the purine ring was substituted at positions 1, 2, 3, 7, 8, or 9 (see Fig. 1), and concluded that Z was an N^6^-substituted adenine.

At Gif, all groups listed in Table 1, except that of Skoog, presented papers, but only one claimed to have isolated a pure active compound. This was Henry Wood who considered he had a pure nicotinamide derivative isolated from plant tumor tissue that caused cell division. This erroneous conclusion arose because of an attempted determination of structure with impure (non-crystalline) products (Letham pers. comm.).

At the conference, Letham met Carlos Miller who was also working with *Z. mays.* Letham writes:

Miller summarised all his data re his maize factor. Miller's factor, my plum factor & my maize factor all had very similar UV spectra, were very probably N^6^-substituted adenines and closely related (or? identical). However, N^6^-substituents could differ in structure.

I suggested to Miller that we collaborate on elucidation of structure, but he declined and wished to move onto other things, e.g. mechanism of kinetin action and rapid responses to cytokinin. When the Z structure was determined and chemically synthesized (first by Shaw in England and soon after in Auckland), he said he was indebted to me for disposing of his “problem”. He got a generous supply of synthetic Z and so did Skoog and others. This was the beginning of a friendship with Miller that lasted until his death.

On my way back to NZ, I visited Skoog in Wisconsin and showed him the slides from my talk, including one of the crystals. He was impressed! He had a big project on isolating pure cytokinin from pea blanch water obtained from a cannery. Just before I arrived, he said they had just held a group meeting to discuss how to further purify their factor—they had come to an impasse and were thinking of trying sublimation! Before I left, after he showed me around & I went to his house for dinner, he said I was welcome to work in his dept!

Skoog's principal worker was a Polish scientist, Rogozinska, supported by three others. Rogozinska et al. (1965) refer to the partial purification of a cell division factor from 6,900 L of pea (*Pisum sativum*) blanch water. In the initial work on pea, Zwar and Skoog (1963) had indicated the presence of more than one bioactive component in extracts from young pea seedlings. One factor was purified to being 1,600 times more active than the starting material but was still 1000 times less active than kinetin. Rogozinska et al. (1965) gained further purification, indicating the factor was at least of similar activity to kinetin in the tobacco (*Nicotiana tabacum*) tissue bioassay, showed that it differed from kinetin, and was likely a purine derivative. We now know that peas have multiple cytokinin forms—nucleotides, ribosides, and free bases, as well as *O*- and *N*-glucosides (Fig. 1)—with the cytokinin at the highest concentration being the nucleotide of isopentenyl adenine (iPRMP) in the seed coats (Grant et al. 2021).

On return to NZ, Letham extracted three lots of 60 kg of maize seeds (harvested about seven days before normal harvest) with ethanol and applied each extract to a very large column (10.2 cm in diameter × 85 cm in length) of sulphonic acid resin ion exchange. Following washing, the sample was eluted with 1.5 M NH_4_OH, and purified with Ag^+^, followed by large-scale paper chromatography as described already. The eluted fraction was precipitated with picric acid. The precipitate was washed and then crystallized. Recrystallization gave a constant melting point of 189–190°C. The picric acid derivative was subjected to anion exchange (acetate form) to yield the free base. This was again crystallized giving pure Z with a constant melting point of 208–209°C. The yield was 0.75 mg from 60 kg of sweet corn. A purification impasse had been overcome by this new approach: firstly, the purification of Z as the picrate by crystallization followed by crystallization of Z. Having crystalline compounds facilitates identification when synthetic compounds are available. If natural and synthetic have the same melting point, and a mixed melting point shows no depression, the identification is unambiguous.

### The structure of zeatin

Determination of the structure of Z was greatly facilitated by fortunate advances in instrumentation. About 1960, two powerful physical methods for structure elucidation became available, namely MS and NMR. However, in 1963 they were not sufficiently sophisticated to handle samples <1 mg. Letham relates how he wrote unsuccessfully to two MS pioneers, Djerassi at Stanford and J.H. Beynon at ICI Manchester, requesting a determination of MS for Z. Then he heard that Jim Shannon, an Australian scientist that he had not met, had returned from the UK and had set up a German Atlas MS at Coal Research (CSIRO) in Sydney in 1961. He was the only person in Australia who determined MS of organic compounds. When contacted, he said he normally required several mg of a sample but if he had a set of model compounds related to the unknown, he would endeavor to do a sub mg spectrum.

Letham relates:

Accordingly, we sent him eight compounds, all adenine derivatives of established purity. Zeatin, our unknown (about 0.5 mg), was placed in a Pregl micro weighing bottle and stoppered. It was all placed in the post with one nagging thought. How good is the chemistry at Coal Research? Do they realise that 0.5 mg was derived from 60,000 grams of plant? However, no need to be concerned! Two to three weeks later I received an envelope in Auckland that contained: a perfect MS of zeatin; an MS of each model compound; an interpretation of the fragmentation pattern of each model compound and of zeatin; an MS of deuterium-labelled zeatin revealing exchangeable hydrogens; and finally there was the only likely chemical structure in either *cis* or *trans* form. Only one word can describe it all—brilliant, and it was all confirmed by another equally distinguished episode. I.R.C. McDonald at Chemistry Division, DSIR, Petone, New Zealand, obtained an NMR of zeatin picrate with only a mg without a time averaging computer using a special microcell he had designed. Both the MS and NMR testified to the purity of the isolated products.

The above data, with some minor chemical degradative studies, formed the basis of the three main publications concerning zeatin structure, namely Letham et al. (1964, 1967) and Shannon and Letham (1966). However, the paper devoted to MS (1966) also contained important additional data—metastable ions in all nine spectra. The metastable ions in the MS of zeatin confirmed the direct formation from zeatin of all ion structures used to assign the zeatin structure. This article, the first concerning MS of N^6^-substituted adenines, is a classic in cytokinin research. All the above data gave an unequivocal structure for zeatin: 6-(4-hydroxy-3-methyl-but-2-enyl)aminopurine, which could be either *cis* or *trans*.

Shannon's technical skill was matched by his ability as a lecturer in MS, describing diverse structures he often elucidated as a service. About 1965 he made a lecturing tour-de-force to New Zealand as a guest of the NZ Institute of Chemistry. He was impressed by my facilities for the tissue culture bioassay which we used to guide purification of the crystals sent to him, and how we tolerated tractors in the floor below!

On one occasion his lecture had important consequences for some cytokinin workers. Prior to publication of the zeatin structure in 1964, the structure was actually shown during an MS lecture by Jim Shannon in Melbourne. He forgot to clean the blackboard! After his lecture, a session of the annual conference of the Australian Society of Plant Physiologists began and the zeatin formula was displayed for the CSIRO cytokinin workers to see. The zeatin structure shown was very probably the key to the unknown apple fruitlet cytokinin being investigated by Kefford and co-workers (Zwar et al. 1963). Kefford wrote to Auckland DSIR for confirmation.

However, these CSIRO workers had stated in *Science* that the cytokinins purified by Miller and by Letham were artifacts (Kefford 1963). This claim was found to be erroneous by Miller (1965) using comparative chromatography of extracts with synthetic Z and its riboside (ZR). Both the synthetic cytokinins were provided by Letham. In this paper, Miller credits Letham with the identification of zeatin. The low activity of the apple fruitlet extract fractions eventually resulted in the cessation of the CSIRO project.

When the structure of Z was established, Miller sent Letham some of his partially purified product. By this time Miller had twice obtained crystalline material from his extracts, but both MS attempts had failed resulting in the loss of all the crystalline material, and thus a portion of an earlier stage of the extractions was sent to Letham (R.M. Amasino, pers comm., 2023).

Letham writes:

It contained only one major UV absorbing compound but was heavily contaminated with other impurities. I completed purification to give crystalline products characterised by melting points etc, and showed it was identical to zeatin. (Letham and Miller 1965)

Miller told Amasino on several occasions what a wonderful person Letham was and how grateful he was that they could publish together in 1965 (R.M. Amasino, pers. comm., 2023). Supplemental File S3 provides further historical information and counters Skoog's claim (Skoog 1994) that Miller should be credited with the “isolation and composition” of Z, a statement with which, as noted earlier, Miller disagreed.

Miller and Witham (1964) commented that substituted amino purines may exist as riboside and nucleotide forms, and later showed in bioassay of chemically, enzymatically, and chromatographically separated maize extract the existence of four active zones suggested to include Z, a nucleoside of Z containing *cis*-hydroxyl groups, and a monophosphate nucleotide of Z (Miller 1965). Subsequently, Letham (1968, 1973) suggested that there was a complex of seven cytokinins in addition to Z in maize, confirming the identity of ZR (Letham 1966b) and ZR-5′-monophosphate (ZR nucleotide, ZRMP) (Letham 1966c), and subsequently purifying both compounds in crystalline form (Letham 1973). The nucleotide was the principal cytokinin in the extracts (7.8 mg from 65 kg of sweet corn). He also showed that the *cis* isomers of these may be present in lesser amounts (Letham 1968).

From the nucleotide fraction, another interesting N^6^-substituted adenine was also purified and crystallized although it showed little cytokinin activity. It was identified unequivocally as the nucleoside of adenylosuccinic acid, an intermediate in AMP biosynthesis not isolated previously from plant tissue (Letham 1973). In this study, 9-glucosylpyranosylzeatin (Z9G) was reported as an endogenous cytokinin in maize (more detail on conjugates is presented later), while evidence for the occurrence of *cis* isomers of ZR and ZRMP was given at the Ottawa Plant Growth Substances conference (Letham 1968). However, some cytokinin activity was reported to be due to N^6^-substituted adenines present at low (sub 200 μg/65 kg maize) levels. These compounds, insufficient to crystallize, appeared to include oxidation products of Z, e.g. 2-hydroxy-Z, and Z with a side chain carrying additional hydroxyl groups (Letham 1973). Such compounds, initially regarded as endogenous, are now known to be artifacts associated with the polystyrene sulphonic acid resin (H^+^ form) when recycled. When replaced by a cellulose phosphate column, the anomalous compounds were not detected (Summons et al. 1980; D.S. Letham, unpublished data).

### Zeatin application to plants and early results

**Figure 3.**
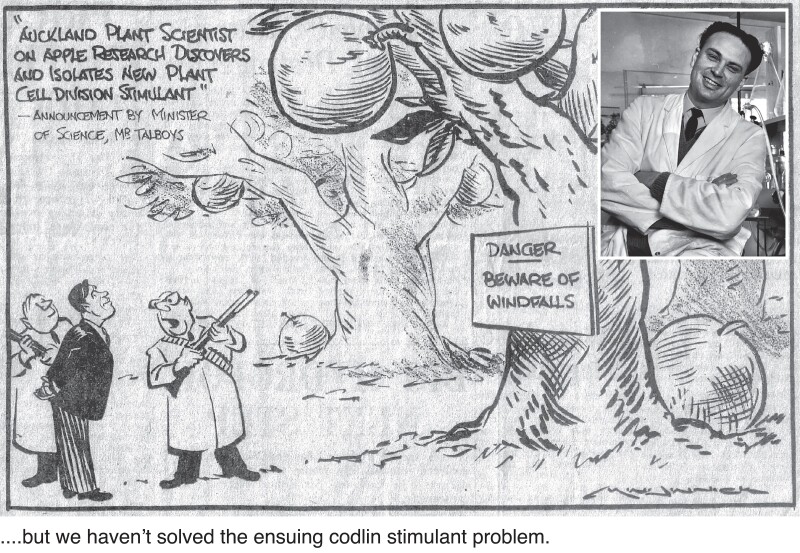
A cartoon published in an Auckland paper in 1964 relating to the identification of zeatin. Insert: Photo of Stuart Letham taken in his lab at DSIR Auckland in 1964. Letham writes: “My pleasant expression was not caused by crystals of Z, but by the reporter from the NZ Herald who took the picture. He brought a cartoon to show me before it appeared in the paper and showed an apple tree with giant fruit (sprayed with zeatin) from which giant codling moths were emerging. Standing next to the tree, armed with a shot gun was the director of FRD (the likeness is striking) shooting down giant moths. The photo shows how well we dressed on a daily basis at that time in the lab. And I worked from 8 am to 6 pm with 10 min for lunch. How times have changed! The official DSIR start time was 8 am sharp”.

Clearly, DSIR Auckland was excited about the identification of Z (Fig. 3) and saw its potential use in preventing postharvest storage disorders, but substantial quantities needed to be synthesized.

Letham writes:

Natural zeatin could occur in *cis* or *trans* forms and stereo-specific chemical synthesis was required to establish this stereo chemistry. Shaw and Wilson (1964) achieved the first synthesis from tiglic acid which at that time was thought to be unambiguous in terms of *trans* and *cis* orientation. It gave a product identical to the natural compound (MS, NMR, chromatography, mixed melting point). However, a more critical examination of the synthesis showed it was really ambiguous stereo chemically (Letham and Young 1971) because *cis-trans* isomerisation occurred during the reaction sequence (conversion of an allylic bromide to azide). Furthermore, the initial synthetic “compound” was a 3 : 2 mixture of methyl γ-bromotiglate and its isomeric ester (Letham and Young 1971). By modification of Shaw's synthesis and by addition of TLC, ^3^H- and ^14^C-zeatin were prepared on a small scale. By crystallization this synthesis can also yield zeatin on a larger scale without defining stereochemistry absolutely.

However, to apply to apple trees, the Auckland workers needed gram amounts of Z of defined *cis-trans* structure. A synthesis was devised and the necessary chemicals obtained. Letham had worked for nine years in Auckland with only one technician to assist and was hoping to attract some postgraduate assistance. Fortuitously, Dr Tony Cebalo, who had recently obtained a PhD in chemistry from the University of Auckland, came to assist with the synthetic work while awaiting a position in the USA. This gave the work access to the equipment of the local Chemistry Department, including IR and NMR, greatly facilitating progress. Cebalo initiated a 6-step synthesis of Z starting with β-methylcrotononitrile which was converted to *trans*-γ-acetoxy-β-methylcrotononitrile (a key stereo intermediate) by bromination followed by reaction with potassium acetate in acetic acid. Continuing on, in six weeks, gram amounts of Z were prepared (Cebalo and Letham 1967), confirming the *trans* structure and providing the new hormone for application to plant systems.

At DSIR Auckland, Z, synthesized as described above, was used to promote cell division by application to developing apple fruitlets (Letham et al. 1969). The resulting developed apples had reduced cell volume, increased tissue density, and altered shape. Thirteen other potential stimulants of cell division, including coconut milk and DPU, did not reduce cell size in the apple fruit (Letham 1969b).

**Figure 4.**
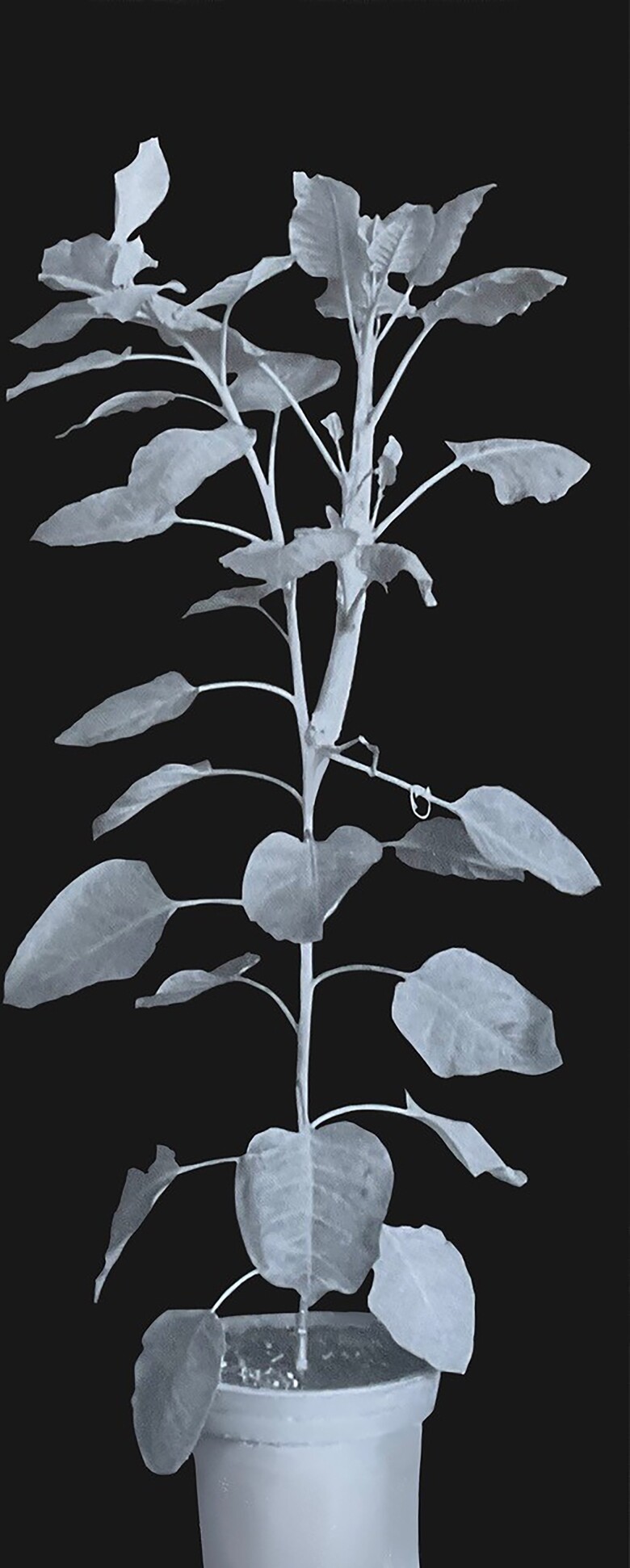
A tobacco plant showing the ability of cytokinin to promote the growth of lateral buds. Zeatin was applied as a lanolin paste (0.5%) to the lateral bud at the base of the petiole bearing the wire loop. This released the bud from apical dominance. Zeatin paste was then applied over the surface of the resulting shoot. A very thick vigorous shoot with developing lateral buds was formed. The photograph was taken 34 d after the start of the experiment. Photo courtesy of L. Engelbrecht.

A number of overseas workers received and used samples in interesting ways. In these early days, L. Engelbrecht (Halle, Germany) revealed the marked ability of Z to release lateral buds from apical dominance (Fig. 4), and Nitsch and Nitsch (1967) used the synthetic Z to induce abundant bud growth of stem segments in tissue culture—something not achieved with any other chemical (Letham 1969c). Miller found Z induced cell division in his soybean tissue culture assay at 10^−11^ M, while 6-(3-methylbut-2-enylamino)purine (isopentenyladenine (iP); Fig. 1), which lacks the allylic hydroxyl group present in Z, required 10^−9^ M (Miller 1967). These requirements were almost identical to those applied to the carrot secondary phloem bioassay used by the workers in Auckland, confirming the potency of Z. Then, using supplied Z and ZR as reference compounds, Miller found conclusively that these compounds were produced by the fungus *Rhizopogon roseolus* (Miller 1967), while iP had been identified in *Corynebacterium* (*Rhodococcus*) *fascians* cultures by Helgeson and Leonard (1966). Thus, the occurrence of cytokinins had been substantially extended by identification in micro-organisms.

In all assays, Z was markedly more active than kinetin in inducing cell division. However, like other cytokinins, Z can, in some situations, also markedly promote cell enlargement as in etiolated bean (*Phaseolus vulgaris*) leaf disks and *Spirodela oligorrhiza* fronds (Letham 1967) and excised radish (*Raphanus sativus*) cotyledons (Letham 1968; Gordon and Letham 1975). The increment in frond area attributable to cell enlargement induced by Z at 0.05 μM was 4 times that caused by iP. Again, the importance of the allylic hydroxyl group is emphasized.

After this early limited work concerning the properties of Z based on synthetic Z prepared by Cebalo and Letham (1967), interest in the synthesis of Z continued (see review by Shaw 1994), although laboratory suppliers began selling Z about 1968. However, synthesis of ^14^C- and ^3^H-Z became important, and a useful synthesis involving interaction between DSIR Auckland and ANU (Young et al. 1990) based on work initiated in 1971 (Letham and Young 1971) merits brief mention. The synthesis also confirmed the *trans* structure of Z and ZR. The starting compound was methyl 3,3-dimethylacrylate which was brominated to yield methyl 4-bromo-3-methyl-crotonate (*cis-trans* mixture). Mild hydrolysis yielded pure trans [E]-methyl-4-hydroxy-3-methyl-crotonate because the *cis*-hydroxy ester underwent facile lactonization and could be readily eliminated. This compound was converted in three steps to (E)-4-(tetrahydropyran-2-yloxy)-3-methylbut-2-enyl bromide. Reaction of the bromide directly with [2-^3^H]adenosine (24 Ci/mmol] and 1- to N^6^ rearrangement yielded ^3^H-labeled ZR. Modification of the reaction yielded ^3^H-Z (Young and Letham 1969). Metabolic studies utilizing labeled Z and ZR are described in later sections.

### Cell division factors in coconut milk

Unlike Letham's research into the physiological factors leading to fruit disorders during storage, early researchers using coconut milk were interested in embryogenesis and organogenesis. Coconut milk had been used since the 1940s to facilitate what was described as “artificial parthenogenesis.” Van Overbeek et al. (1941) used coconut milk to facilitate the growth of pro-embryos in vitro. Notably, roots did not develop until the embryos were removed from the medium containing coconut milk, which was ascribed, at the time, to the presence of a heat-stable inhibitor, possibly auxin (Van Overbeek et al. 1941, 1942). It is now well recognized that cytokinin inhibits root growth in vitro.

Steward was also working on embryogenesis and using coconut milk that clearly contained a cell division-inducing factor. They reported that cell division in carrot root phloem tissues was increased by the addition of coconut milk (Caplin and Steward 1948). This response was then utilized as a bioassay (Caplin and Steward 1949, 1952; Steward et al. 1952). Van Overbeek in 1941, followed by Steward (Caplin and Steward 1948), and Skoog with five co-workers (Mauney et al. 1952) all endeavored to identify growth factors in coconut milk (Table 1). No endogenous cytokinin was purified.

A hurricane in Florida had brought down thousands of coconuts and Steward hired a tanker and filled it with coconut milk—providing over 800 gallons of coconut milk to work with. Shantz and Steward (1952) acknowledged that “Access to the large bulk of coconut milk was made possible through the generous help of the Grasseild Chemicals Division of the du Pont Co.” They reported that the growth-promoting activity of coconut milk was due to at least three distinct entities which they had isolated in crystalline form. One compound was active in the bioassay at 20 ppm (Shantz and Steward 1952). A story, not that dissimilar to that of kinetin, relates to the identification of the first cell division-inducing factor isolated from coconut milk, also published in 1955. This factor was identified as *N*,*N*′-diphenyl urea (DPU) (Fig. 1) (Shantz and Steward 1955). Unfortunately, as related in Amasino (2005) “to prepare this large batch, commercial equipment at a Dupont plant was used. Steward's group did not know that this equipment had been previously used for preparing diphenylurea herbicides.” While DPU is likely to have been a contaminant it is weakly active biologically in its own right (reviewed in Shudo 1994). Synthetic derivatives of DPU, such as CPPU and thidiazuron, are now used commercially (Shudo 1994). They are more active than DPU, which is less active than kinetin.

Meanwhile, Ted Bollard had spent some time in Steward's lab during 1957–1958 working on nitrogen metabolism and xylem sap N. He had a lengthy discussion about his ideas with Steward which subsequently appeared in a review written by Steward, with no acknowledgment to Bollard. Bollard was incensed when later told by a Cornell confidant that Steward had taped their conversation. An angry Bollard said to Letham around 1962 that it would be nice to isolate the active ingredient from coconut milk. Letham writes:

Just before leaving for the GIF conference, Ted (Bollard) said last thing: take careful note of how far Steward has got with his coconut milk; is he purifying a natural cell division factor? Reply at once, cable if possible, as it would be 3 weeks before I returned to FRD.

Steward's most active fractions were active at ppm, about 3 mg/L. Remembering Z was active at <0.2 ug/l (in the same assay), I advised Ted that F.C.S [Steward] had probably a long way to go. He may need to concentrate his activity 10,000 times (needs some very specific steps!). When I saw Ted later he said: “our orchard staff have been busy working to help you after the structure of zeatin is finished. In the deep freeze there is about 100 L of coconut milk (from 500 hundred coconuts)”. Such was Ted's enthusiasm to solve the coconut question.

In 1967, at the Ottawa Plant Growth Substance meeting, Letham reported the bioassay-guided purification of ZR obtaining 0.4 mg crystalline ZR from 60 L of coconut milk (Letham 1968)—this included mixed melting-point determinations and MS. Further detail is provided in Letham (1974), where it is clear that there were biologically active components in addition to ZR.

In addition to Steward's and Letham's groups, a number of others were also detecting biologically active components in coconut (see Letham 1974). Clearly, there was more than one source of biological activity and 11 cytokinins have now been identified from coconut milk. Van Staden identified Z and ZR—suggesting these were present in roughly equal quantities (Van Staden and Drewes 1975)—and *O*-glucosylzeatin (ZOG) (van Staden 1976a).

Kobayashi et al. (1995, 1997) isolated and identified a highly water-soluble compound active in the tobacco callus bioassay. 14-O-(3-O-[β-D-galactopyranosyl-(1→2)-α-D-galactopyranosyl-(1→3)-α-L-arabinofuranosyl]-4-O-(α-L-arabinofuranosyl)-β-D-galactopyranosyl)-*trans*-zeatin riboside [G3A2-ZR]. This compound was more active than DPU but less active than ZR. They considered it contributed about 20% of the cytokinin-active components in coconut milk, while not detecting Z, ZR, or DPU in their extracts. They also emphasized the fact that while Z and ZR are sparingly water-soluble, G3A2-ZR was highly water soluble as befits a biologically active compound present in the liquid endosperm of coconut and which could be metabolized to Z.

In addition to G3A2-ZR, 10 cytokinins have been unequivocally identified and quantified in the endosperm of young, green coconuts (Yong et al. 2009). The greatest quantity was of ZR followed by ZOG, dihydrozeatin-OG (DHZOG), ZRMP, and oT, with traces of kinetin, KR, iP, DHZ, and Z. The reported identification of both kinetin and kinetin riboside in trace amounts in coconut milk may well be related to the degradation of DNA as suggested by Barciszewski et al. (1997).

Coconut milk has the remarkable ability of being able to induce cell division in many species of plant tissue in culture, including quiescent tissue such as carrot root secondary phloem. The bioassay-guided purification of ZR as a principal cytokinin of coconut milk in 1967, and the ability of cytokinins to substitute for coconut milk in the above cultures, together established that cell division induced by this fluid is basically a response to cytokinin. Thus, 10 μl/L Z was substituted for coconut milk (10% by volume, 6.7 g total solids/L) in carrot phloem cultures, while in early work kinetin (250 μg/L) was substituted for coconut milk in apple and other pome-fruit cultures (Letham 1958b, 1960b, 1964).

## Cytokinins and tRNA

Letham and Ralph (1967) and Hall et al. (1967) reported almost simultaneously that hydrolysates of plant soluble RNA (s-RNA) contained a ZR-like cytokinin. By MS, Hall identified this as *cis*-ZR and a new chapter of the zeatin story began. *Cis*-Z was synthesized by Leonard et al. (1971) who showed that *cis*-Z was over 50 times less active in the tobacco callus bioassay than *trans*-Z. *Cis*-Z cytokinins supplied exogenously are not converted to the *trans*-isomers in plant tissues (Palni et al. 1985; Wang et al. 1997a; Nogué et al. 2000), so endogenous *cis*-Z does not appear to be a route to *trans*-Z.

The presence and release of *cis*-derivatives of Z from tRNA, detection of these as free cytokinins in extracts of various plants, and their low reactivity in bioassays, led to a debate about their biological relevance (see references in Parker et al. 1989). Indeed, Tay et al. (1986) suggested they were derived by enzymatic hydrolysis of tRNA during extraction. To help resolve this controversy, Parker et al. (1989) developed radioimmunoassay (RIA) for quantifying *cis*-Z and *cis*-ZR using antibodies raised against *cis*-ZR and applied this to xylem sap samples, which avoided the initial extraction phase when the formation of free cytokinins could occur. Notably, RIA of the samples only took place following substantial purification and HPLC. Both *c*ZR and *c*Z were detected as components of xylem sap of wheat (*Triticum aestivum*) and oat (*Avena sativa*), albeit at lesser levels than *trans*-isomers, thus confirming the presence of *cis*-derivatives as free cytokinins.

The presence of cytokinins in specific positions of plant tRNA molecules and the debates surrounding the relevance of such cytokinins, particularly the *cis*-Z isomer in free form, are covered in e.g. Kaminek (2015) and Hluska et al. (2021). It should also be noted that tRNA-specific isopentenyl transferase (IPT) genes have been found in organisms ranging from archaea to humans (references in Spíchal 2012; Wang et al. 2020), thus indicating that cytokinins are likely to be found in all life forms.

## Beyond zeatin: metabolism and conjugation

### Identification of conjugates

In 1967 the MS world changed—the high-resolution AEI MS9 appeared—mg almost became μg in sample size and MW was accurate to four decimal places. By 1983, multiple conjugates of Z and 6-benzylamino purine (BA; Fig. 1) had been identified (Letham and Palni 1983). For example, when ^3^H-Z was applied to de-rooted radish seedlings, multiple metabolites were detected leading to the isolation and identification of the 7- and 9-glucosides of Z (Z7G, Z9G). Zeatin riboside 5′-phosphate (ZRMP; Fig. 1) was the major peak after 3 h incubation but, following 8 h incubation and 15 h in water, the major metabolite was Z7G, named raphanatin (Parker et al. 1972, 1973; Gordon et al. 1974). When maize seedlings were exposed to ^3^H-Z for 20 h, Z, ZR, and ZRMP were detected. However, the major metabolite in the excised roots of maize was identified as the Z9G, but this metabolite was not seen when de-rooted maize plants were used (Parker et al. 1973).

In further experiments with radish using ^3^H-BA, two glucosides were detected as the major metabolites: these were subsequently identified as the 7- and 9-β-D-glucopyranosides of BA (Parker et al. 1973; Wilson et al. 1974; Cowley et al. 1978). Additionally, 6-benzylamino-3-β-D-glucopyranosylpurine was identified as a minor metabolite (Letham et al. 1975; Parker et al. 1975).

The 7- and 9-glucopyranoside conjugates of Z have now been isolated from diverse plant species and were synthesized by unambiguous methods in Letham's lab (Parker et al. 1973, 1975; Duke et al. 1975; Summons et al. 1977, 1979a). The 3-, 7- and 9-glucosides are particularly unusual, and were the first compounds isolated from plants with a glucose moiety attached to an *N*-atom in a purine ring. The 7-glucosides are unusual in the site of the glycosidic linkage and in the sugar involved. Certain compounds related to vitamin B_12_ were the only previously known natural purines with a sugar (in these cases ribose) at position 7, while the cytokinin 3-, 7-, and 9-glucosides were the first purine glucosides found in nature. The 7- and 9-glucosides of Z and BA were synthesized in both the pyranose and furanose forms (Cowley et al. 1978) and this enabled the natural glucosides to be assigned β-D-pyranoside structures (MacLeod et al. 1976) and not the furanoside structure as proposed by Fox and co-workers (Deleuze et al. 1972) based on erroneous interpretations of mass spectra.

The 3-glucoside of BA was markedly more active than the 7- and 9-glucosides in several bioassays (Letham et al. 1983), and is rapidly metabolized releasing BA (Letham and Gollnow 1985). Because of their low activity in bioassays, the 7- and 9-glucosides of Z have generally been considered to be inactivated conjugates (Letham et al. 1983; van Staden and Drewes 1991). This hypothesis is supported by the metabolic stability of the [^3^H]-Z9G (Nogué et al. 2000) and with earlier studies on the stability of labeled 7- and 9-glucosides (Gawer et al. 1977; Palni et al. 1984; Letham and Gollnow 1985). However, there are recent claims, summarized in Chen et al. (2021), that suggest both *t*Z7G and *t*Z9G could potentially contribute to the pool of active cytokinins, whereas the iP-*N*-glucosides are likely terminal metabolites (Hallmark et al. 2020; Hošek et al. 2020).

An interesting side story relating to these unusual metabolites is provided in Supplemental File S4, where the synthesis of enzyme inhibitors of 7- and 9-glucosylation by Letham led to the development of the cancer-inhibiting drug “roscovitine.” In relation to human cancer cells, it is noteworthy also, that cytokinin ribosides, but not the free bases, are very effective inhibitors of cell growth (Voller et al. 2010).

Simultaneously with the identification of the 7- and 9-glucosides, a second set of Z conjugates, in which the glucose moiety is attached to the N^6^ side chain, was also identified (Parker et al. 1975; Letham et al. 1977). Metabolism of ^3^H-Z in blue lupin (*Lupinus angustifolius*) leaves led to the unequivocal identification of *O*-β-D-glucopyranosylzeatin (ZOG) (Parker et al. 1978), a compound equally active as Z in three bioassays (Letham et al. 1983). It was clear from the work in a number of labs, based on β-glucosidase treatment, chromatography, and bioassays, that *O*-glucoside conjugates were widespread across plant species, being detected by several groups during the 1970s, including in poplar (*Populus × robusta*) (Hewett and Wareing 1973), soybean (Horgan 1975), and weeping willow (*Salix babylonica*) (van Staden 1976b), and identified in crown gall (Peterson and Miller 1977; Morris 1977). Letham's group confirmed that poplar species metabolized ^3^H-Z and ^3^H-ZR to a complex of cytokinins including four *O*-glucosides—ZOG, DHZOG, ZROG, and DHZROG—each of which was chemically synthesized and unequivocally identified as endogenous cytokinins (Letham et al. 1977; Duke et al. 1979; Summons et al. 1979b).

Because of the ease of release of the free bases and ribosides from the *O*-glucosides (e.g. Letham and Gollnow 1985), these have generally been regarded as storage compounds, readily turned over to release base or riboside, and hence their activity in bioassays (Letham et al. 1983).

Additional novel conjugates of Z were identified in blue lupin. L-β-[6-(4-hydroxy-3-methylbut-trans-2-enylamino)-purin-9-yl]alanine was the only plant product known in which an amino acid moiety is conjugated to a nitrogen atom of the purine ring. This weakly active metabolite was named lupinic acid (Parker et al. 1975, 1978; Duke et al. 1978). Supplied lupinic acid was barely metabolized compared to ZOG in lupin leaves (Parker et al. 1978), suggesting it is a stabilized but inactivated conjugate. The weak activity of lupinic acid observed in the soybean callus bioassay was attributable to the trace amounts of released Z detected (Palni et al. 1984).

A comparison of metabolism in xylem/pith tissues with that in bark/phloem tissues of blue lupin indicated that metabolism was rapid and complex, and led to the identification of four *O*-acetyl derivatives in bark tissues: *O*-acetyl-9-β-D-ribofuranosyldihydrozeatin 5′-monophosphate (AcDHZRNT) and AcZRNT (Letham and Zhang 1989), and AcZR and AcDHZR (Zhang et al. 2002). Both methylated Z and DHZ metabolites also appeared to be in stem bark and in excised pod walls (Zhang et al. 2002). Acetylation appeared to reduce breakdown to adenine-type compounds (Zhang et al. 2002) and, like ZOG, AcZ was active in bioassays (Letham 1978), while the methylated forms showed low activity (Letham 1972). Little further investigation seems to have been done with either the methylated or acetylated metabolites, although ZR was shown to be converted to AcZR in tobacco tumor tissue (Mornet and Laloue 1989), and acetylated Z and ZR were detected as endogenous forms following chilling of daffodil (*Narcissus pseudonarcissus*) bulbs (Letham et al. 2003).

### Translocation and metabolism studies

The synthesis of ^3^H- and ^14^C-Z (Letham and Young 1971) facilitated the investigation not only of metabolism but also of the movement of cytokinins throughout the plant. Sondheimer and Tzou (1971) may have been the first to publish metabolism of Z: they supplied [8-^14^C]Z to bean axes and detected radioactivity in Z and DHZ [i.e. 6-(4-hydroxy-3-methylbutyl- amino)purine] (DHZ; Fig. 1) and in their ribosides and 5′-nucleotides. However, differential metabolism between species was soon found by the Letham lab, and the principal metabolite of Z in radish cotyledons was Z7G, the compound named raphanatin (Parker et al. 1972). Differential metabolism within organs of a plant and between species is clearly a feature of the cytokinins (reviewed in Jameson 1994).

Letham's development of 2-D silica gel TLC, normal and also reversed-phase TLC (Letham et al. 1992) for use following feeding of labeled cytokinins, facilitated in-depth metabolic studies (e.g. Badenoch-Jones et al. 1984b, 1984c; Palni et al. 1984; Jameson et al. 1987). Location of metabolites was validated using synthetic standards within each system.

Many papers show movement and metabolism of applied cytokinins throughout the plant, with cytokinin ribosides highlighted as the translocated form in the xylem. An early example showed that ^3^H-ZR was the only form of ^3^H in the xylem sap when ^3^H-Z was applied to radish roots, but when ^3^H-Z was applied to cotyledons ZR was absent and Z7G predominated (Gordon et al. 1974). Although the long-distance transport of cytokinins as “carriers of information from roots to shoots” was challenged by Faiss et al. (1997), cytokinins clearly are highly mobile and can act as systemic signals (summarized in Sakakibara 2021). A variety of cytokinins, including ribosides, free bases, and lesser quantities of nucleotides and occasionally *O*-glucosides have been detected in the xylem of, for example, blue lupin (Jameson et al. 1987), soybean (Noodén et al. 1990), *N. rustuca* (Singh et al. 1992a), mistletoes (*Amyema* species), and their *Eucalyptus* hosts (Hall et al. 1987), and in the xylem exudates of pigeon pea (*Cajanus cajan*) plants nodulated by a leaf-curl-inducing rhizobium strain (Upadhyaya et al. 1991a). The ribosides (ZR and DHZR) are generally, but not always, in greater quantity [See also later discussion of the involvement of both ZR and Z as systemic signals].

### Quantification of cytokinins

#### Deuterated cytokinins

Studies of cytokinin function and physiology really required precise mass spectral quantification of cytokinins but accurate quantification of cytokinins in plant tissues was limited until the production of stable deuterated derivatives. To achieve this, Letham collaborated with Roger Summons, John MacLeod, and Colin Duke from the Research School of Chemistry at ANU, who labeled every major natural cytokinin in the isoprenoid side chain with deuterium (Summons et al. 1977). Use of these standards enabled Z-type cytokinins to be accurately quantified in plant tissues (maize kernels, radish, and yellow lupin (*Lupinus luteus*) seed) for the first time (Summons et al. 1977, 1979a, 1979b). Then, to provide a basis for studies of seed germination, Hocart and Summons quantified 12 endogenous cytokinins in dry maize seed including free bases and ribosides, *O*-glucosides, 7- and 9-glucosides, and nucleotides (Hocart et al. 1988).

The synthetic routes of deuterated derivatives are detailed in Summons et al. (1979a) and outlined in Entsch et al. (1980). This substantial contribution from the Letham lab was taken up commercially by Apex Organics (England) who were bought out by Olchemim Ltd (Olomouc, Czech Republic). Present-day quantification by the highly sensitive LC-MS/MS techniques utilizes these deuterated cytokinins as internal standards.

Critically, extraction, purification, and separation of the various cytokinin forms are necessary first steps in both chemical and biological quantitation of endogenous cytokinins (Letham 1978). Moreover, endogenous cytokinins exist in plant tissues in pmol to fmol levels. At these levels, it is impossible to quantify samples based on UV absorbance during HPLC separation because of UV-absorbing contaminants. As noted by Mildaziene et al. (2022), many of the publications on seeds treated with non-thermal plasma are based on inaccurately measured hormone levels, including those of Z.

#### Inhibition of phosphatases

Early metabolic studies showed rapid conversion of ^3^H-Z and ZR to nucleotides, and their subsequent turnover. Consequently, inhibition of phosphatase activity during extraction is critical as the cytokinin nucleotides are readily broken down by phosphatases to release the ribosides. A modification of the method developed by Bieleski for extraction of phosphate esters after phosphatase inactivation (Bieleski 1964) is widely used and is referred to in the cytokinin literature as Bieleski's solvent. First use of this solvent in cytokinin extractions is described in the metabolic studies by Parker et al. (1972), Parker and Letham (1973), and Gordon et al. (1974). By carefully preventing phosphatase action, the nucleotides were shown to be very early metabolites following the supply of Z or ZR to plant tissues. The importance of these metabolites was also recognized by others (e.g. Laloue et al. 1974).

Moreover, it is now recognized that the formation of the cytokinin nucleotides is the first committed step in the biosynthetic pathway catalyzed by IPT (Kakimoto 2001; Takei et al. 2001). As the cytokinin nucleotides are converted directly by LONELY GUY (LOG) to the active cytokinin-free bases (Kurakawa et al. 2007; Kuroha et al. 2009; Chen et al. 2022), their retention intact should be informative. If the nucleotides are degraded during extraction to release cytokinin ribosides, the nucleotides will be under-estimated and the ribosides over-estimated in samples.

#### Radioimmunoassay

During the 1980s, many labs were developing RIAs for cytokinins (see references in Badenoch-Jones et al. 1987). The sensitivity of RIAs often filled the gap before the development of modern LC-MS/MS. The Letham lab also developed antibodies for use in RIA and used their knowledge of the comprehensive array of cytokinin moieties to assess the efficacy and ease of use of immunoassays. Badenoch-Jones et al. (1984a, 1987) provide a comprehensive analysis of the pros and cons of RIA and provide details of the extensive pre-purification that is an absolute requirement prior to immunoassay, which contrasted markedly to Weiler's suggestion of utility with crude extracts (Weiler 1980). The necessity for such pre-purification and separation of the nucleotides, free bases, ribosides, 7- and 9-glucosides is because of cross-reactivity with the antibodies: these moieties cross-react with the relevant anti-riboside antibody, while the *O*-glucosides do not cross-react. Moreover, there are many compounds within plant (and animal) cells with isopentenyl moieties that might also cross-react with anti-iPR serum (Badenoch-Jones et al. 1987). As Badenoch-Jones et al. (1984a) stated “Generally, the amounts of each cross-reactive compound in a crude extract are unknown and hence the result on a crude extract may not even be a good indicator of the total amount of the cross-reactive cytokinins. Furthermore, the activity of a compound in RIA is determined by its structure and may bear little relation to its biological activity.”

Commercially, preprepared plates are available for ELISA. However, the necessity for pre-purification and separation of individual cytokinins eludes many of the more recent users of commercial ELISA kits, leading, in some cases, to the cytokinin content of a sample being presented simply as “cytokinin” (e.g. Tsago et al. 2020), solely as Z (e.g. Wu et al. 2020) or as the ribosides of Z, iP, and DHZ (e.g. Wang et al. 2019). In Tsago et al. (2020) the antibody is not identified, and in none of these examples was any reference made to cross-reactivity of the free bases and ribosides, or glucosides for that matter, with the relevant antibody.

## Contributions to plant development and physiology

This section introduces some of the early discoveries that pointed to the critical role of cytokinins in plant physiology and development. The classical definition of a cytokinin is based on its ability to stimulate cell division of plant tissues grown in vitro, i.e. in callus bioassays, in the presence of an auxin (Horgan 1984). However, it was clear that the role of cytokinins *in planta* was not necessarily restricted to cell division.

### Senescence

Cytokinins have been associated with the delay of senescence since the early work of Richmond and Lang (1957). Both metabolism studies and measurements of endogenous cytokinins in various types of senescence strengthened this association. For example, Letham and Gollnow (1985) showed that senescent and non-senescent radish cotyledons differed in their metabolism of Z, ZR, and BA. Instead of metabolizing Z and ZR to the 7-glucoside, there was enhanced metabolism to adenine derivatives in senescent cotyledons, and little metabolism to form the 3-glucoside of BAP, compared with non-senescent cotyledons. In assessing sequential leaf senescence of *Nicotiana rustica*, a marked decline in Z levels was associated with senescence, and pre-senescent leaves possessed cytokinin levels which were two to eight times those of senescent leaves near the stem base (Singh et al. 1992a). Singh et al. (1992b) suggested that a difference in cytokinin biosynthetic capacity may contribute to the differing cytokinin levels in leaves of different maturity and may participate in the control of sequential leaf senescence.

The relationship between podfill, monocarpic senescence, and cytokinins was investigated in soybeans. Here, ZR, DHZR, Z, and DHZ in the xylem sap dropped sharply during early pod development to levels below those expected to retard senescence. However, an increase in xylem sap cytokinins (particularly ZR and DHZR), occurred if pods at full extension were removed, a process that also delayed senescence. Depodding at late podfill, which did not delay senescence, did not increase the cytokinin levels greatly (Noodén et al. 1990). Noodén and Letham (1993) suggested that modulation of cytokinin flux into the leaf appears to be a sensitive signaling mechanism controlling the senescence of leaves and other parts.

### Rhizobium and nodulation

An investigation into the role played by auxins and cytokinins in the initiation, development, and maintenance of root nodules led to the first unequivocal identification of IAA produced by *Rhizobium trifolii* in culture supernatants, and the conclusion that IAA is not involved in the root curling process, a process that is a precursor to nodule initiation (Badenoch-Jones et al. 1982). While nodules were clearly able to metabolize ZR (Badenoch-Jones et al. 1984b), they concluded that the defect in nitrogen fixation in ineffective nodules was not associated with a major alteration in cytokinin metabolism (Badenoch-Jones et al. 1984c).

The impact of endogenously produced, xylem-mobile cytokinins was investigated using an unusual *Rhizobium* strain. The leaf curl syndrome of pigeon pea was shown to be a systemic response to effective nodulation by *Rhizobium* strain IC3342 (Upadhyaya et al. 1991b). Features of the leaf curl syndrome include the release of apical dominance, lateral shoot proliferation, hyponasty, and leaf curling (Upadhyaya et al. 1991a, 1991b). Elevated cytokinin activity was detected in the sap and leaf extracts derived from leaf curl plants when compared to those obtained from normal plants and symptomless Curl^−^ mutants. Notably, there was a predominance of ZR and DHZR in bleeding sap whereas in culture the free bases, iP and Z, predominated (Upadhyaya et al. 1991a, 1991c), giving further support to the ribosides as the predominant transport form. RIA data were confirmed by GC-MS and provided the first unequivocal identifications of cytokinins produced by *Rhizobium* bacteria (Upadhyaya et al. 1991a, 1991c). In an interesting extension of this work, Yong et al. (2014) provided elevated nitrogen which suppressed both *N*-fixation and development of the syndrome and showed, on the removal of *N*, the xylem cytokinin levels increased and the leaf curl syndrome was induced. This included the release of apical dominance and, additionally, increased leaf thickness and increased stem diameter, leading to the conclusion that xylem sap cytokinin acts as a pleiotropic regulator of plant development (Yong et al. 2014). In this work, an elegant version of a root pressure chamber was used to collect xylem sap from a single cut petiole while the transpiration rate of the whole plant was maintained unchanged allowing the xylem sap cytokinins entering the leaf blade and foliar cytokinins to be compared concurrently (Yong et al. 2000).

### Altered meristem program (*amp1*) mutant

Until the discovery of genes for cytokinin biosynthesis and metabolism, our ability to manipulate cytokinin levels relied on exogenous applications, as cytokinin mutants were rare. However, the *amp1* mutant of Arabidopsis (*Arabidopsis thaliana*) was an exception. It was shown to have five times more cytokinin than the wild type (Chaudhury et al. 1993). The elevated cytokinin, which was a consequence of increased biosynthesis (Nogué et al. 2000), led to decreased apical dominance, increased life span, and increased regeneration into vegetative shoots (from roots); the *amp* mutants also exhibited de-etiolation in the dark, often had increased cotyledon number and an increased rate of leaf formation. They exhibited precocious flowering but had reduced fertility (Chaudhury et al. 1993; Nogué et al. 2000). The pleiotropic effect of elevated cytokinin is again obvious.

### Transgenic plants containing the bacterial *ipt* gene

The identification of an *ipt* gene from the T-DNA of *Agrobacterium tumefaciens* (see review in Morris 1986) allowed investigations into the interaction between cytokinin and auxin. Zhang et al. (1995) showed the level of cytokinin was greatly elevated in the transgenic tobacco tissue expressing the native *ipt* gene, with shoots forming on the calli independent of cytokinin and auxin. However, the cytokinin level was reduced by exogenous auxin. They showed auxin not only enhanced the activity of cytokinin oxidase (CKX) in vitro and promoted the activity of both glycosylated and non-glycosylated forms of CKX (Zhang et al. 1995) but also reduced the level of both IPT mRNA and protein (Zhang et al. 1996). Addition of BA reversed the negative effect of auxin (Zhang et al. 1996).

Another important contribution from studying cytokinin biosynthesis in transgenic tobacco tissues indicated that the *trans*-hydroxylation of iP-type cytokinins to yield Z-type cytokinins occurred principally at the nucleotide level, as opposed to at the riboside or free base level (Zhang et al. 1995), a finding subsequently validated by Takei et al. (2004) who identified the relevant gene.

In order to better control the cytokinin levels and to regenerate whole plants, a chimeric cytokinin biosynthetic gene was utilized (Wang et al. 1997a, 1997b). This was constructed by placing the coding region of the bacterial *ipt* gene under the control of a chalcone synthase (CHS) promoter (P_CHS_) from *Antirrhinum majus*. The P_CHS_-*ipt* gene was transferred to tobacco. The control was a P_CHS_-GUS construct which showed that GUS expression was, contrary to expectations, not limited to petals. Expression of the *ipt* gene elevated the cytokinin level in mature leaf laminae which was associated with retardation of leaf senescence and increased rates of transpiration due to changes in number, size, and aperture of stomata; in the upper stems of flowering plants elevated cytokinin was associated with the development of lateral shoots, but also the development of larger and broader leaves, enlargement of midribs and veins of leaves, an increase in node number of primary stems, and promotion of cell division in internodes. Generally, stems were thicker and root growth reduced. As little as a 2-fold elevation in endogenous cytokinin level caused clear changes in development (Wang et al. 1997a, 1997b). Supporters of sensitivity to plant hormones as the controlling factor in development (a popular view in the 1980s) had claimed that such changes were too small to be physiologically relevant (Trewavas 1982), which is clearly not the case here.

Additionally, using the transgenic tobacco tissues, *cis*-Z was shown to be a substrate for CKX, and there was no conversion of *cis*-Z to (*trans*)-Z. Moreover, elevated endogenous cytokinin promoted CKX activity and the rate of degradation of exogenous ZR to adenosine was enhanced (Wang et al. 1997a, 1997b), an important aspect of cytokinin homeostasis noted by Motyka et al. (1996), and subsequently widely observed.

The P_CHS_-*ipt* gene was also transferred into potato (*Solanum tuberosum*) (Tao et al. 2010). The transgenic plants had elevated Z-type cytokinins, similar morphological changes as the transgenic tobacco, along with branched aerial stolons, many terminating in microtubers. The number of underground tubers was enhanced but their weight reduced relative to controls potentially due to a redirection of assimilates to above-ground parts, due to the increased stem size (Tao et al. 2010). The very large increase in cell number observed in IPT stems and the elevated cytokinin level, particularly in the subapical and apical tissues caused by P_CHS_-*ipt* gene expression, were both positively associated with increased CDK activity in the subapical stem segment. Clearly, many of the effects of cytokinin are due to enhancement of cell division.

A much more tightly regulated promoter—that of the seed-specific *vicilin* gene from pea (Higgins et al. 1988)—was also used. The chimeric *vicilin-ipt* was introduced into tobacco cells (Ma et al. 1998). The expression of the *vicilin-ipt* was shown to be confined to the seed and resulted in enhanced levels of cytokinins in the developing seeds and increased seed protein content. The growth of the transgenic plants and the development of the seeds appeared otherwise normal.

## Turn of the century

Molecular biology tools and the publication of the Arabidopsis genome led to a surge in cytokinin gene discovery by workers predominantly in the USA and Japan. In 1999, a number of genes coding for the metabolic enzymes were reported. The gene coding for *O*-glucosyltransferase, the enzyme that forms ZOG from Z, was cloned from *Phaseolus lunatus* seeds (Martin et al. 1999). A gene encoding a maize cytokinin oxidase (CKX), which cleaves the N^6^ -side chain of cytokinin bases and ribosides to yield adenine and adenosine respectively, was cloned (Houba-Hérin et al. 1999; Morris et al. 1999). Finally, in 2001, it was confirmed that plants have the ability to biosynthesize their own cytokinins via an IPT. Two Japanese labs showed that the plant IPT exists as a small gene family in Arabidopsis (Kakimoto 2001; Takei et al. 2001) and prefers ADP and ATP as precursors, in contrast to the *ipt* gene of *A. tumefaciens* that prefers AMP. Takei et al. (2004) subsequently identified the gene (*CYP735A*) coding for the enzyme that *trans*-hydroxylates iPRMP to *trans*-ZRMP, thus forming the precursor of Z.

Also in 2001, cytokinin molecules were shown to be detected by receptor molecules of a similar type to the ethylene receptors. In Arabidopsis, the cytokinin signal was shown to be perceived by three sensor histidine kinases (HK), AHK2, AHK3, and CYTOKININ RESPONSE1 (CRE1)/AHK4 (Inoue et al. 2001; Suzuki et al. 2001; Ueguchi et al. 2001; Yamada et al. 2001). The perceived signal is transduced via a 2-component signaling circuit (Hwang and Sheen 2001; Hwang et al. 2002, 2012) leading to phosphorylation of response regulators (view Romanov et al. (2018) for a model of the signal transduction pathway).

A lack of cytokinin-deficient plants was overcome by the production of transgenic plants over-expressing CKX (Werner et al. 2001, 2003). The cytokinin-deficient plants developed stunted shoots with smaller apical meristems. The plastochrone was prolonged, and leaf cell production was only 3% to 4% of that of the wild type, indicating an absolute requirement of cytokinins for leaf growth. In contrast, root meristems of transgenic plants were enlarged and gave rise to faster growing and more branched roots. These results showed that cytokinins are an important regulatory factor of plant meristem activity and morphogenesis, with opposing roles in shoots and roots. Functional studies, this time using receptor mutants confirmed the pleiotropic role of the cytokinins in plant growth and development (Riefler et al. 2006). Further, ectopic expression in transgenic Arabidopsis of *ARABIDOPSIS RESPONSE REGULATOR 2* (*ARR2*), the rate-limiting factor in the response to cytokinin, was shown to be sufficient to mimic cytokinin in promoting shoot meristem proliferation and leaf differentiation, and in delaying leaf senescence (Hwang and Sheen 2001), again confirming the multiple roles of cytokinins in plants.

The free bases (Z, *cis*-Z, DHZ, and iP; Fig. 1) were confirmed as the active forms (Lomin et al. 2015; Romanov and Schmülling 2021). Relevant here is the recent challenge to ZR as merely a transport form and the suggestion by Nguyen et al. (2021) that ZR is an active form. Both Romanov and Schmülling (2021) and Chen et al. (2022) challenged this proposal stating it is unlikely that ZR is an active form per se, as it is unlikely to bind with sufficient efficacy to cytokinin receptors.

Furthermore, grafting experiments by both Osugi et al. (2017) and Landrein et al. (2018) showed ZR, produced outside the meristem, operating as a systemic signal but, notably, ZR could not trigger the cytokinin response within the stem cells of the shoot apical meristem. The systemic signal (ZR) is reliant on conversion to the nucleotide and the presence of LOG enzymes within the meristem for the nucleotide to be locally converted to active cytokinin (i.e. Z) in the shoot meristem. Additionally, however, Osugi et al. (2017) suggested that the Z detected in the xylem may also act as a systemic signal relating to leaf size. This work, elaborated in the recent model shown by Sakakibara (2021), confirms the critical role of xylem-mobile cytokinins.

## An ancient molecule essential for life on earth

Zeatin and related natural compounds occur in every species of flowering plant to control and coordinate growth and development. Their presence is an absolute requirement for leaf growth and, as such, cytokinins are essential for life on earth. However, as Letham et al. (2019) noted, the function of cytokinins extends far beyond plants. For example, Z is produced by certain human microbial pathogens that also respond to applied cytokinin. This was shown with an obligate protozoan pathogen (Andrabi et al. 2018) and with *Mycobacterium tuberculosis* which occurs naturally only in humans (Samanovic et al. 2015, 2018). *M. tuberculosis,* several other human pathogens, other bacteria, and even archaea have been shown to contain homologs of LOG, the plant cytokinin activating enzyme (see references in Letham et al. 2019; Chen et al. 2022). Much remains to be elucidated regarding the role of cytokinins in such organisms.

Zeatin has one other remarkable feature not mentioned thus far. It is an ancient molecule present at the dawn of aerobic life on Earth. Zeatin is synthesized in cyanobacteria (Frébortová et al. 2015), the organisms which oxygenated the atmosphere of Earth in the Great Oxidation Event that initiated some 2,400 million years ago (Gumsley et al. 2017). The cytokinin biosynthetic genes appear to have been transferred from cyanobacteria to plants by horizontal gene transfer initiated by endosymbiosis of cyanobacteria giving rise to chloroplasts (Spíchal 2012).

## Concluding remarks

As set out above, it is incontrovertibly the case that Letham and co-workers provided the first unequivocal identification of Z in 1964. In 1985, I was introduced to Folke Skoog at a conference by one of the delegates who added that I was from New Zealand. Professor Skoog's immediate response was “more's the pity.” Being somewhat taken aback I simply asked why. Skoog's response was “Letham was from New Zealand and he stole Miller's data.” In further discussion, Skoog agreed with me that Letham had subsequently made a great contribution to cytokinin biology (something Skoog repeated in his 1994 article!). Having now spoken with Letham, as have both Professors Miroslav Kaminek (Czech Republic) and Thomas Schmülling (Germany) (see Kaminek 2015), and received written documentation from Letham (including the transcribed comments in Supplemental File S3), and comments from Richard Amasino (USA), it is clear that Letham did not steal Miller's data. It is highly significant that Miller sent his partially purified sweet corn factor to Letham, who further purified it, identified it, and showed that it was identical to zeatin, co-authoring the subsequent paper (Letham and Miller 1965).

Alongside his contributions to cytokinin biology over the last 60 years, Letham has witnessed the evolving importance of cytokinins. A postscript on Letham's letter to me of May 7th 2018 read: “The significance of zeatin seems to be ever increasing—now *Mycobacterium tuberculosis*. Its detection in Cyanobacterium suggests CK were here at the dawn of life.”

## Supplementary Material

kiad094_Supplementary_Data

## Supplemental data

The following materials are available in the online version of this article.

**Supplemental File S1**. Miller CO, Witham FH. Régulateurs de la Croissance Vegétalé conference in Gif-sur-Yvette, France July 1963; published as an Errata as it was omitted from the GIF proceedings that were published in 1964 (S1A: clean copy; S1B: copy annotated by Letham).

**Supplemental File S2**. Letham (1964). Régulateurs de la Croissance Vegétalé conference in Gif-sur-Yvette, France, July 1963; GIF proceedings published 1964.

**Supplemental File S3**. Which lab first identified zeatin? A controversy invented by Folke Skoog.

**Supplemental File S4**. Inhibitors of cytokinin glucosyl transferases: From zeatin and radish cotyledons to cancer treatment: the story of roscovitine.
