# Supplementary material for: Zeatin: The 60th anniversary of its identification
Source: Plant Physiol. 2023 Feb 15;192(1):34–55. doi: 10.1093/plphys/kiad094 (PMC10152681; doi:10.1093/plphys/kiad094)
Supplement: kiad094_Supplementary_Data [file kiad094_supplementary_data.zip › suppfileS1.pdf]

Supplemental File S1A

A KINETIN-LIKE FACTOR FROM MAIZE AND OTHER SOURCES (1)

C. O. Miller and F. H. Witham
Indiana University, Bloomington, Indiana, U. S. A.

Résumé

Un facteur de croissance ressemblant à la kinétine a été extrait par l'éthanol de caryopses de maïs, purifié d'abord sur colonnes échangeuses d'ions, puis par chromatographie sur papier. La fraction active a pu être sublimée sous vide mais avec de grandes pertes.

Le spectre U.V. du composé obtenu est essentiellement le même que celui de la kinétine. Ses deux pKa sont 2,5 et 11,0 environ. Le facteur actif ne donne pas la réaction avec la cystéine-SO_4H_2 que donne la kinétine. Il ne passe pas dans l'éther comme la kinétine et a une plus grande activité biologique sur le test utilisé (cultures de cal de soja). Le facteur actif est probablement une adénine substituée en position 6 avec une chaîne comportant un radical —OH.

Les racines, feuilles et tiges, inflorescences et fruits du maïs contiennent tous des substances actives sur le test soja. Il en est de même des jeunes fruits d'*Helianthus annuus*.

For many years various investigators have recognized the immature grain of maize as a good source of plant growth substances and a brief summary of work with the material has been presented by Steward and Shantz [6]. More recently, Beauchesne [1] has neatly confirmed that several different types of growth substances are present in the grains. In Beauchesne's experiments one group of substances behaved as cations and promoted cell division in the tobacco pith test. It is apparently with a substance from this group that we have been concerned in our own research.

This particular substance was selected for study because its behaviour both in growth tests and isolation procedures made it seem likely it is related chemically to kinetin. In fact, the purification procedure [4] was modeled after that previously used in the isolation of kinetin [3]. The growth assay used, however, is a more recently developed one in which a soybean callus tissue shows an almost absolute requirement for a kinetin-type of compound for any sort of growth and even for continuance of life [5].

(1) Research supported by a grant from the National Science Foundation.

Isolation

A summary of the isolation method follows. To purify the factor, a 70% ethanol extract of maize kernels was run through a column of the strongly acid cation exchanger Dowex 50 (H^+) ; the factor stuck to the column, although considerable material active in the growth assay came through. After a water wash, the factor was eluted from the column with 6*N* NH_4OH. After evaporation of the ammonia from the eluate, acidification to pH 1.5, and removal of the solids which formed, the solution was run through a smaller column of the same resin. The column was developed sequentially with water, 1.5 *N* HCl and then with 4 *N* HCl ; the compound came off soon after the first of the 4 *N* HCl had been applied. The active fractions were pooled, diluted to about 1 *N* HCl and put back onto a Dowex 50 (H^+) column which was subsequently washed with water and then with 6 *N* NH_4OH, the factor being eluted with the latter. The ammonia in turn was removed under vacuum. The compound was further purified by paper chromatography with several different solvent systems. The four most useful systems and the approximate Rf values obtained in ascending chromatography on Whatman No. 1 filter paper were :

1) *t*-butanol (3) + H_2O (1) + conc. NH_4OH (1) : 0.82 ;
2) *n*-butanol (2) + benzene (1) + H_2O (1) + methanol (1) : 0.75 ;
3) 1 *N* NH_4OH : 0.72 ;
4) water-saturated *n*-butanol : 0.61.

The active material thus obtained could be sublimed under vacuum but very considerable loss was experienced when we attempted to achieve final purification by this technique with a large amount of the compound.

We estimate that we have obtained anywhere from 0.03 to 0.1 milligram of factor from each kilogram of milky-stage corn kernels.

Some properties.

The compound obtained in a rather clean condition shows ultraviolet absorption spectra essentially the same as those exhibited by kinetin, both having peaks at 268-269 millimicra in ethanol and peaks around 273 or 274 millimicra in acid or base. Both compounds have two obvious pKa values, those of the factor being at approximately 2.5 and 11.0. The maize factor, however, does not give a color reaction with cysteine-H_2SO_4 and does not go into ether as does kinetin ; the factor is also more easily dissolved in water. The two can be separated by paper chromatography although they run fairly close together in our systems. The purified factor is more sensitive to mineral acids than is kinetin and shows a reaction to acetic anhydride whereas kinetin does not. If one assumes that kinetin and the factor have the same molar extinction coefficients in ultraviolet light, it is then possible to make growth comparisons at various concentrations. On such an assumption it seems that the factor from maize has a higher level of activity at very low concentrations. Thus, our data support the conclusion that the factor considerably promotes growth at a concentration of only 8.8×10^{-9} Molar whereas kinetin has little effect at this concentration (Table I). At much higher levels, the concentration curves tend to run parallel.

Table I

RESPONSE OF SOYBEAN CALLUS TISSUE TO KINETIN AND FACTOR FROM MAIZE

Concentration of Compound (1) (millimicromoles/liter)	Fresh Weight of Tissue (2) (mg/piece)		
	Control	Kinetin	Factor
0	13	—	—
8.8	—	12	150
88.0	—	71	287
176.0	—	145	405

(1) Concentration of the factor prepared on the assumption that the factor and kinetin have the same molar extinction coefficients in ultraviolet light ; this may not be valid.
(2) Average of sixteen pieces ; 23 days growth.

Analysis of the compound.

Since the compound acted in the same manner as kinetin in the major isolation steps, exhibited the same absorption spectra, and gave the same growth effect, we naturally thought that it is probably a 6-(substituted)amino-purine. All of the information we have at the moment supports this idea.

If a portion of the factor is treated at room temperature with a neutral solution of potassium permanganate at a concentration of 1 mg/l, two new compounds immediately appear. Comparisons of these compounds with known substances as to movement in several chromatographic setups and as to absorption spectra in the ultraviolet lead us to believe that they are adenine and probably N-(purin-6-yl)glycine. This suggests that the factor is adenine substituted in the amino group. Our studies of the spectra strengthen this belief. Since the absorption peak in ultraviolet light shifts to longer wavelengths when the pH is raised from neutrality to alkaline values, it seems that the purine ring is not substituted on the 7 or 9 positions. Also, the wavelength of minimal absorption in ultraviolet shifts to a higher value as the pH is changed from 1 to 7 ; therefore, it is likely that the substituent is not attached to position 3 [2]. The spectra and stability of the compound in base make substitution on the 1 position seem unlikely. We have not been able with certainty to rule out possible substitutions on the 2 and 8 carbons ; the ease with which both adenine and the glycinyl derivatives are obtained, however, seems to mitigate against such possibilities.

If one assumes that the compound is adenine with the amino group substituted, the interest then shifts to the nature of the substituent. The second carbon of the

substituent apparently is subject to attack by the potassium permanganate and this may mean a double bond between the second and a third carbon. However, this position in the factor seems more easily oxidizable to produce the glycinyl derivative than the same position in model compounds such as 6-allylaminopurine. Therefore, we suspect that there may be additional substitution on the second carbon ; the presence of an oxygen atom, for example, would increase the possibility of production of the observed product of the permanganate reaction. Unlike kinetin or 6-(alkyl)amino-purines, the factor reacts quickly with acetic anhydride or more slowly with glacial acetic acid to produce a derivative which moves more easily in our chromatographic systems. This derivative may be changed back to the factor by heating in 0.01 *N* HCL at 100° C. This suggests the derivative may be an ester of the factor, and that the factor may possess an OH group or another group which can give rise to it. This interpretation is supported by the presence in the nuclear magnetic resonance spectrum of a peak very likely attributable to a group in which an oxygen atom is attached to a carbon atom. It is even conceivable that this same oxygen is on the second carbon. Perhaps it is significant that kinetin itself comes close to meeting the apparent structural requirements of the isolated compound.

Natural occurrence

One must consider the possibility that the compound we have studied might be made during the isolation process. We known that this particular compound represents only a small fraction of the total activity in the original maize extract, but we chose to study it because it has many properties in common with kinetin. We have thought that if this compound truly is naturally occuring, then derivatives of it such as nucleosides, nucleotides or even more complicated ones probably exist. It is conceivable the compound is produced during isolation from these more complicated active molecules of which it is a part. Or perhaps the factor is actually made from an inactive compound during isolation. We have made some attempts to check the latter possibility. Elimination of alcohol and ammonium hydroxide from the purification methods did not alter the results. When we chromatographed ethanolic extracts of kernels without any use of acid or cation exchange resins, we obtained most of the activity at low R_f values but also substantial activity at the higher R_f values where we would expect the factor to be. In addition, we have obtained no activity by running deoxyadenosine through the isolation procedure. We certainly do not feel confident that we are working with a naturally occuring compound, but neither do we feel sure that it is not at least a part of the natural active substance. Obviously, it is highly desirable that someone develop an entirely different method of isolation so that results from different procedures may be compared.

Distribution in the plant.

If the factor or a close active derivative does occur naturally, then both the distribution in the plant and the time of presence should be of interest in studies of growth and development. Therefore, we have analyzed for the substance in the parts

of the plant at different ages. During the first stages of purification we often did not detect activity from these plant parts but did detect activity after the material had been put through the last column. However, in all such cases we observed a very definite toxicity due to the cruder preparations. After elution from the last column, we have found good activity in preparations from stems and leaves of all ages, young roots (we haven't analyzed old roots), tassels, cobs, kernels, and unpollinated ears (Table II). In terms of amount of the factor per gram of dried plant part, the yield from the kernels was highest although all parts had substantial amounts. It

TABLE II

RESPONSE OF SOYBEAN CALLUS TISSUE TO MATERIAL ISOLATED FROM PARTS OF MAIZE PLANTS OF VARIOUS AGES (1)

Age of Plant (weeks, days)	Part of Plant	Fresh Weight of Soybean Tissue (mg/piece) (2)
4,0	Roots	199
	Stems and leaves	40
6,0	Roots	129
	Stems and leaves	78
8,0	Stems and leaves	233
	Tassels	396
8,5	Young ears	161
	Tassels	183
9,4	Kernels (milky)	897
	Cobs	651
10,2	Kernels	857
	Cobs	272
10,5	Stems and leaves	550
11,0	Unfertlized ears	456
	Control, no kinetin	2
	Control, 0.5 mg/l kinetin	816

(1) Material from 50 grams (dry weight) of plant part included in 1 liter basal medium for each part of plant tested.
(2) Average of 12 pieces, 28 days of growth.

may be of interest that the yield from stems and leaves consistently increased throughout the growing season.

Occurrence in other plants.

Using the same isolation procedures described earlier, we have obtained preparations from young sunflower fruits which show the same absorption peaks in ultraviolet light, the same movements on paper chromatograms and the same type of growth effect as the substance from maize. Also, we have purified a compound from soybean protein enzymatic hydrolysate which has identical properties. Probably either the compound or material from which it is made is widespread in plants.

Literature Cited

[1] Beauchesne G. (1961). Séparation des substances de croissance d'extrait de maïs immature. *In* : Plant Growth Regulation. Iowa State University Press, Ames, Iowa, p. 667-674.

[2] Leonard N. J. and Deyrup J. A. (1962). The chemistry of triacanthine. *Jour. Amer. Chem. Soc., 84,* 2148-2160.

[3] Miller C. O., Skoog F., Von Saltza M. H. and Strong F. M. (1955). Kinetin, a cell division factor from deoxyribonucleic acid. *Jour. Amer. Chem. Soc., 77,* 1392.

[4] Miller C. O. (1961). A kinetin-like compound in maize. Proc. Nat. Acad. Sci. (U.S.A.), *47,* 170-174.

[5] Miller C. O. (1963). Kinetin and kinetin-like compounds. *In* : Modern Methods of Plant Analysis *VI,* 194-202.

[6] Steward F. C. and Shantz E. M. (1959). The chemical regulation of growth (some substances and extracts which induce growth and morphogenesis). *Ann. Rev. Plant Physiol., 10,* 379-404.

Supplemental File S1B. Annotations by D.S. Letham

A KINETIN-LIKE FACTOR FROM MAIZE AND OTHER SOURCES (1)

C. O. Miller and F. H. Witham
Indiana University, Bloomington, Indiana, U. S. A.

Résumé

Un facteur de croissance ressemblant à la kinétine a été extrait par l'éthanol de caryopses de maïs, purifié d'abord sur colonnes échangeuses d'ions, puis par chromatographie sur papier. La fraction active a pu être sublimée sous vide mais avec de grandes pertes.

Le spectre U.V. du composé obtenu est essentiellement le même que celui de la kinétine. Ses deux pKa sont 2,5 et 11,0 environ. Le facteur actif ne donne pas la réaction avec la cystéine-SO_4H_2 que donne la kinétine. Il ne passe pas dans l'éther comme la kinétine et a une plus grande activité biologique sur le test utilisé (cultures de cal de soja). Le facteur actif est probablement une adénine substituée en position 6 avec une chaîne comportant un radical —OH.

Les racines, feuilles et tiges, inflorescences et fruits du maïs contiennent tous des substances actives sur le test soja. Il en est de même des jeunes fruits d'*Helianthus annuus*.

For many years various investigators have recognized the immature grain of maize as a good source of plant growth substances and a brief summary of work with the material has been presented by Steward and Shantz [6]. More recently, Beauchesne [1] has neatly confirmed that several different types of growth substances are present in the grains. In Beauchesne's experiments one group of substances behaved as cations and promoted cell division in the tobacco pith test. It is apparently with a substance from this group that we have been concerned in our own research.

This particular substance was selected for study because its behaviour both in growth tests and isolation procedures made it seem likely it is related chemically to kinetin. In fact, the purification procedure [4] was modeled after that previously used in the isolation of kinetin [3]. The growth assay used, however, is a more recently developed one in which a soybean callus tissue shows an almost absolute requirement for a kinetin-type of compound for any sort of growth and even for continuance of life [5].

(1) Research supported by a grant from the National Science Foundation.

Isolation

A summary of the isolation method follows. To purify the factor, a 70% ethanol extract of maize kernels was run through a column of the strongly acid cation exchanger Dowex 50 (H^+) ; the factor stuck to the column, although considerable material active in the growth assay came through. After a water wash, the factor was eluted from the column with 6*N* NH_4OH. After evaporation of the ammonia from the eluate, acidification to pH 1.5, and removal of the solids which formed, the solution was run through a smaller column of the same resin. The column was developed sequentially with water, 1.5 *N* HCl and then with 4 *N* HCl ; the compound came off soon after the first of the 4 *N* HCl had been applied. The active fractions were pooled, diluted to about 1 *N* HCl and put back onto a Dowex 50 (H^+) column which was subsequently washed with water and then with 6 *N* NH_4OH, the factor being eluted with the latter. The ammonia in turn was removed under vacuum. The compound was further purified by paper chromatography with several different solvent systems. The four most useful systems and the approximate Rf values obtained in ascending chromatography on Whatman No. 1 filter paper were :

1) *t*-butanol (3) + H_2O (1) + conc. NH_4OH (1) : 0.82 ;
2) *n*-butanol (2) + benzene (1) + H_2O (1) + methanol (1) : 0.75 ;
3) 1 *N* NH_4OH : 0.72 ;
4) water-saturated *n*-butanol : 0.61.

The active material thus obtained could be sublimed under vacuum but very considerable loss was experienced when we attempted to achieve final purification by this technique with a large amount of the compound.

We estimate that we have obtained anywhere from 0.03 to 0.1 milligram of factor from each kilogram of milky-stage corn kernels.

Some properties.

The compound obtained in a rather clean condition shows ultraviolet absorption spectra essentially the same as those exhibited by kinetin, both having peaks at 268-269 millimicra in ethanol and peaks around 273 or 274 millimicra in acid or base. Both compounds have two obvious pKa values, those of the factor being at approximately 2.5 and 11.0. The maize factor, however, does not give a color reaction with cysteine-H_2SO_4 and does not go into ether as does kinetin ; the factor is also more easily dissolved in water. The two can be separated by paper chromatography although they run fairly close together in our systems. The purified factor is more sensitive to mineral acids than is kinetin and shows a reaction to acetic anhydride whereas kinetin does not. If one assumes that kinetin and the factor have the same molar extinction coefficients in ultraviolet light, it is then possible to make growth comparisons at various concentrations. On such an assumption it seems that the factor from maize has a higher level of activity at very low concentrations. Thus, our data support the conclusion that the factor considerably promotes growth at a concentration of only 8.8×10^{-9} Molar whereas kinetin has little effect at this concentration (Table I). At much higher levels, the concentration curves tend to run parallel.

This product is not pure - it would be contaminated by material derived from the paper and would contain some DZ which also occurs in sweet corn. Crystalline compound, recrystallized to constant melting point, was required for determination of structure. Mixed melting points can then be made with synthetic compounds. This was important in determining the *trans* structure of zeatin

TABLE I

RESPONSE OF SOYBEAN CALLUS TISSUE TO KINETIN AND FACTOR FROM MAIZE

Concentration of Compound (1) (millimicromoles/liter)	Fresh Weight of Tissue (2) (mg/piece)		
	Control	Kinetin	Factor
0	13	—	—
8.8	—	12	150
88.0	—	71	287
176.0	—	145	405

(1) Concentration of the factor prepared on the assumption that the factor and kinetin have the same molar extinction coefficients in ultraviolet light ; this may not be valid.
(2) Average of sixteen pieces ; 23 days growth.

Analysis of the compound.

Since the compound acted in the same manner as kinetin in the major isolation steps, exhibited the same absorption spectra, and gave the same growth effect, we naturally thought that it is probably a 6-(substituted)amino-purine. All of the information we have at the moment supports this idea.

If a portion of the factor is treated at room temperature with a neutral solution of potassium permanganate at a concentration of 1 mg/l, two new compounds immediately appear. Comparisons of these compounds with known substances as to movement in several chromatographic setups and as to absorption spectra in the ultraviolet lead us to believe that they are adenine and probably N-(purin-6-yl)glycine. This suggests that the factor is adenine substituted in the amino group. Our studies of the spectra strengthen this belief. Since the absorption peak in ultraviolet light shifts to longer wavelengths when the pH is raised from neutrality to alkaline values, it seems that the purine ring is not substituted on the 7 or 9 positions. Also, the wavelength of minimal absorption in ultraviolet shifts to a higher value as the pH is changed from 1 to 7 ; therefore, it is likely that the substituent is not attached to position 3 [2]. The spectra and stability of the compound in base make substitution on the 1 position seem unlikely. We have not been able with certainty to rule out possible substitutions on the 2 and 8 carbons ; the ease with which both adenine and the glycinyl derivatives are obtained, however, seems to mitigate against such possibilities.

If one assumes that the compound is adenine with the amino group substituted, the interest then shifts to the nature of the substituent. The second carbon of the

This does not establish the presence of a double bond. An OH on each of the 2nd & 3rd carbons would also make the position oxidisable by permanganate (a diol is an intermediate in the oxidation of a double bond to a-COOH)

substituent apparently is subject to attack by the potassium permanganate and this may mean a double bond between the second and a third carbon. However, this position in the factor seems more easily oxidizable to produce the glycinyl derivative than the same position in model compounds such as 6-allylaminopurine. Therefore, we suspect that there may be additional substitution on the second carbon ; the presence of an oxygen atom, for example, would increase the possibility of production of the observed product of the permanganate reaction. Unlike kinetin or 6-(alkyl)amino-purines, the factor reacts quickly with acetic anhydride or more slowly with glacial acetic acid to produce a derivative which moves more easily in our chromatographic systems. This derivative may be changed back to the factor by heating in 0.01 N HCL at 100° C. This suggests the derivative may be an ester of the factor, and that the factor may possess an OH group or another group which can give rise to it. This interpretation is supported by the presence in the nuclear magnetic resonance spectrum of a peak very likely attributable to a group in which an oxygen atom is attached to a carbon atom. It is even conceivable that this same oxygen is on the second carbon. Perhaps it is significant that kinetin itself comes close to meeting the apparent structural requirements of the isolated compound.

The presence of an amino group would also explain results

The CH_2 adjacent to the NH of adenine would give a very similar signal (as we found in nmr of zeatin).

Natural occurrence

One must consider the possibility that the compound we have studied might be made during the isolation process. We known that this particular compound represents only a small fraction of the total activity in the original maize extract, but we chose to study it because it has many properties in common with kinetin. We have thought that if this compound truly is naturally occuring, then derivatives of it such as nucleosides, nucleotides or even more complicated ones probably exist. It is conceivable the compound is produced during isolation from these more complicated active molecules of which it is a part. Or perhaps the factor is actually made from an inactive compound during isolation. We have made some attempts to check the latter possibility. Elimination of alcohol and ammonium hydroxide from the purification methods did not alter the results. When we chromatographed ethanolic extracts of kernels without any use of acid or cation exchange resins, we obtained most of the activity at low R_f values but also substantial activity at the higher R_f values where we would expect the factor to be. In addition, we have obtained no activity by running deoxyadenosine through the isolation procedure. We certainly do not feel confident that we are working with a naturally occuring compound, but neither do we feel sure that it is not at least a part of the natural active substance. Obviously, it is highly desirable that someone develop an entirely different method of isolation so that results from different procedures may be compared.

Distribution in the plant.

If the factor or a close active derivative does occur naturally, then both the distribution in the plant and the time of presence should be of interest in studies of growth and development. Therefore, we have analyzed for the substance in the parts

of the plant at different ages. During the first stages of purification we often did not detect activity from these plant parts but did detect activity after the material had been put through the last column. However, in all such cases we observed a very definite toxicity due to the cruder preparations. After elution from the last column, we have found good activity in preparations from stems and leaves of all ages, young roots (we haven't analyzed old roots), tassels, cobs, kernels, and unpollinated ears (Table II). In terms of amount of the factor per gram of dried plant part, the yield from the kernels was highest although all parts had substantial amounts. It

TABLE II

RESPONSE OF SOYBEAN CALLUS TISSUE TO MATERIAL ISOLATED FROM PARTS OF MAIZE PLANTS OF VARIOUS AGES (1)

Age of Plant (weeks, days)	Part of Plant	Fresh Weight of Soybean Tissue (mg/piece) (2)
4,0	Roots	199
	Stems and leaves	40
6,0	Roots	129
	Stems and leaves	78
8,0	Stems and leaves	233
	Tassels	396
8,5	Young ears	161
	Tassels	183
9,4	Kernels (milky)	897
	Cobs	651
10,2	Kernels	857
	Cobs	272
10,5	Stems and leaves	550
11,0	Unfertlized ears	456
	Control, no kinetin	2
	Control, 0.5 mg/l kinetin	816

(1) Material from 50 grams (dry weight) of plant part included in 1 liter basal medium for each part of plant tested.
(2) Average of 12 pieces, 28 days of growth.

may be of interest that the yield from stems and leaves consistently increased throughout the growing season.

Occurrence in other plants.

Using the same isolation procedures described earlier, we have obtained preparations from young sunflower fruits which show the same absorption peaks in ultraviolet light, the same movements on paper chromatograms and the same type of growth effect as the substance from maize. Also, we have purified a compound from soybean protein enzymatic hydrolysate which has identical properties. Probably either the compound or material from which it is made is widespread in plants.

Literature Cited

[1] Beauchesne G. (1961). Séparation des substances de croissance d'extrait de maïs immature. *In* : Plant Growth Regulation. Iowa State University Press, Ames, Iowa, p. 667-674.

[2] Leonard N. J. and Deyrup J. A. (1962). The chemistry of triacanthine. *Jour. Amer. Chem. Soc.*, *84*, 2148-2160.

[3] Miller C. O., Skoog F., Von Saltza M. H. and Strong F. M. (1955). Kinetin, a cell division factor from deoxyribonucleic acid. *Jour. Amer. Chem. Soc.*, *77*, 1392.

[4] Miller C. O. (1961). A kinetin-like compound in maize. Proc. Nat. Acad. Sci. (U.S.A.), *47*, 170-174.

[5] Miller C. O. (1963). Kinetin and kinetin-like compounds. *In* : Modern Methods of Plant Analysis *VI*, 194-202.

[6] Steward F. C. and Shantz E. M. (1959). The chemical regulation of growth (some substances and extracts which induce growth and morphogenesis). *Ann. Rev. Plant Physiol.*, *10*, 379-404.
