# Supplementary material for: Zeatin: The 60th anniversary of its identification
Source: Plant Physiol. 2023 Feb 15;192(1):34–55. doi: 10.1093/plphys/kiad094 (PMC10152681; doi:10.1093/plphys/kiad094)
Supplement: kiad094_Supplementary_Data [file kiad094_supplementary_data.zip › Supplemental File S2. Letham DS 1964_.pdf]

Supplemental File S2

COLLOQUES INTERNATIONAUX
DU
CENTRE NATIONAL DE LA RECHERCHE SCIENTIFIQUE

N° 123

RÉGULATEURS NATURELS DE LA CROISSANCE VÉGÉTALE

GIF s/YVETTE
15-20 Juillet 1963

EXTRAIT

ÉDITIONS DU CENTRE NATIONAL DE LA RECHERCHE SCIENTIFIQUE
15, QUAI ANATOLE-FRANCE, PARIS (VIIe)
1964

ISOLATION OF A KININ FROM PLUM FRUITLETS AND OTHER TISSUES

D. S. Letham

Fruit Research Division, Department of Scientific and Industrial Research, Auckland, New Zealand

Résumé

Deux facteurs agissant synergiquement sur la division cellullaire d'explantats de carotte ont été purifiés à partir de prunes immatures. L'un de ces facteurs a été cristallisé et identifié au *myo*-inositol. L'autre facteur est une base qui absorbe fortement dans l'ultraviolet. Il produit la division cellulaire en présence d'acide indolyl-3-acétique.

Une substance identique à ce dernier facteur a été isolée et cristallisée à partir de graines de maïs immatures. Cette substance a été nommée « zéatine ». La zéatine est plus active que la kinétine et stimule la division cellulaire dès la concentration de 0,1 μg/1. L'étude des produits de dégradation, des données spectroscopiques et des valeurs du pK pour les groupements dissociables de la molécule indique que la zéatine est une adénine substituée en position N_6.

Factors which induce cell division, and which do not appear to be auxins or gibberellins, have been detected in several plant tissues [4, 10, 13, 14, 15, 16]. Work in this laboratory [7] has shown that fruitlets of a number of species contain factors of this type.

The purification and properties of cell-division stimulants occurring in plum fruitlets are now described. The isolation of one of these factors from immature sweet corn seeds is also reported. Extracts and fractions were assayed for cell-division-promoting activity by determining their ability to promote division in carrot secondary phloem explants in liquid culture [3], growth increments being determined after 21 days. The basal medium contained indole-3-acetic acid (IAA, 2 mg./1.) and has been described in detail [5].

Purification and Properties od Cell division Stimulants in Plum Fruitlet Extracts

Plum fruitlets (diameter 8-15 mm.) were extracted with ethanol at room temperature and the resulting extracts were evaporated *in vacuo* at 40° C. The residue was extracted with water to give an inactive water-insoluble and an active water-soluble fraction (WS). The isolation of the stimulants in WS was complicated by the presence of inhibitors. These were first detected when attempts were made to purify the stimulants by adsorption on and elution from de-activated charcoal. Inhibitory eluates were obtained. To separate the stimulants from the inhibitors, an aqueous solution of WS at pH 3.3 was shaken with ethyl acetate ; the resulting aqueous phase (fraction AP) showed enhanced division-promoting activity while the ethyl acetate extracts (fraction EP) inhibited growth. The effect of these fractions and of WS on the growth of carrot explants is shown in Figure 1. It is seen that the increase in fresh weight induced by WS was slight. The increase in cell number was, however, much greater being about 50 per cent of that given by coconut milk (10 per cent by volume). The increment in cell number induced by AP approached that given by coconut milk. Fraction EP retarded cell enlargement to a greater degree than cell division. Fractions AP and EP in combination produced a growth increment approximately equal to that given by WS.

Properties of factors in AP. — The stimulative and inhibitory activity in AP and EP did not appear to be an artefact of the extraction procedure or of autoclaving for media sterilization.

The activity of AP in normal basal medium and the activity in basal medium lacking IAA were compared. In the absence of IAA, AP produced a small increment in weight but a considerable increment in cell number. In the presence of IAA, both increments were greatly increased, a marked synergism existing between AP and IAA.

The activity of AP fractions derived from plum fruits at different stages of development were compared. Activity reached a maximum about the time of onset of active cell division in the fruit (Fig. 2 and 3). The factors in AP could be responsible for the observed induction of rapid cell division in the fruit.

Separation of factors in AP. — Butanol-extraction studies showed that fraction AP contained at least two synergistic division-promoting factors. These were conveniently separated by ion-exchange methods as next described. AP was passed through a column of 'Zeo-Karb' 225 (H form) and the effluent through a column of either 'De-Acidite' G or Amberlite IR 4B (free base form). No activity was eluted from the weakly basic columns. The 'Zeo-Karb' 225 was eluted in turn with 70 per cent ethanol, 1.5 N NH_4OH, 0.3 N HCl, 1.5 N HCl and 6 N HCl. Activity was detected in the fractions eluted by 1.5 N NH_4OH (fraction A), 1.5 N HCl and 6 N HCl. These fractions interacted synergistically with the fraction (fraction B) not absorbed on the basic columns to promote cell division.

Isolation of the active factor in fraction B. — To isolate the factor, fraction B was chromatographed on sheets of washed seed-test paper and the active zone was eluted with water. After further purification to remove a polyphenolic impurity, the factor

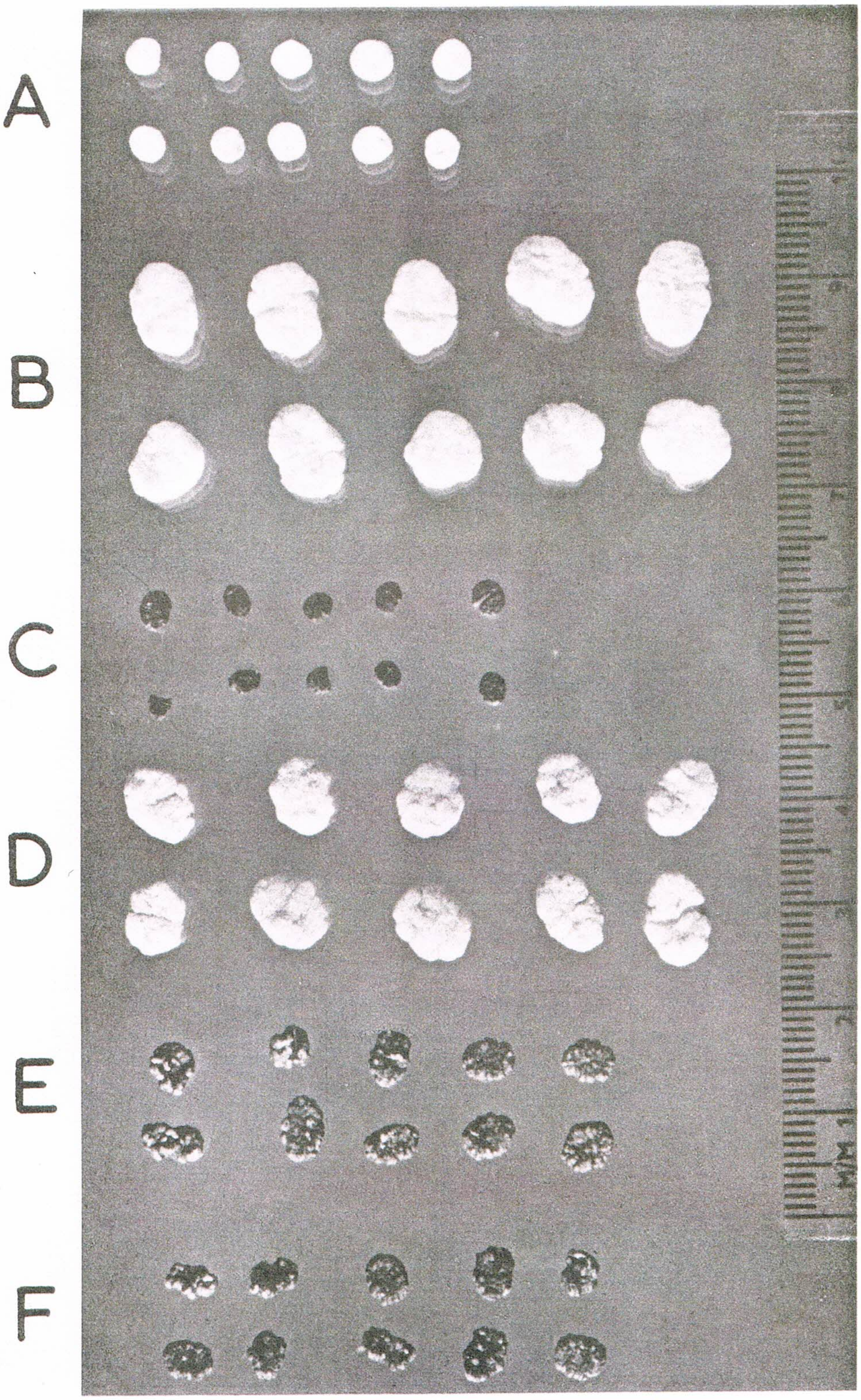

Fig. 1 — Carrot explants cultured on basal medium (A) and on basal medium containing coconut milk (B), fraction EP (C), AP (D), EP + AP (E) and WS (F). Coconut milk was at 10 per cent by volume. The concentration of all plum fractions was the equivalent of 1 g. of tissue per 100 ml. of medium.

was isolated in crystalline form and termed factor B which at 10 mg./l. accounted for all the activity of fraction B. The R_F of factor B in several solvents, its melting point (m. p.) and mixed m. p. with *myo*-inositol, and the m.p. of the acetyl derivative alone and when mixed with *myo*-inositol hexaacetate established that factor B was *myo*-inositol.

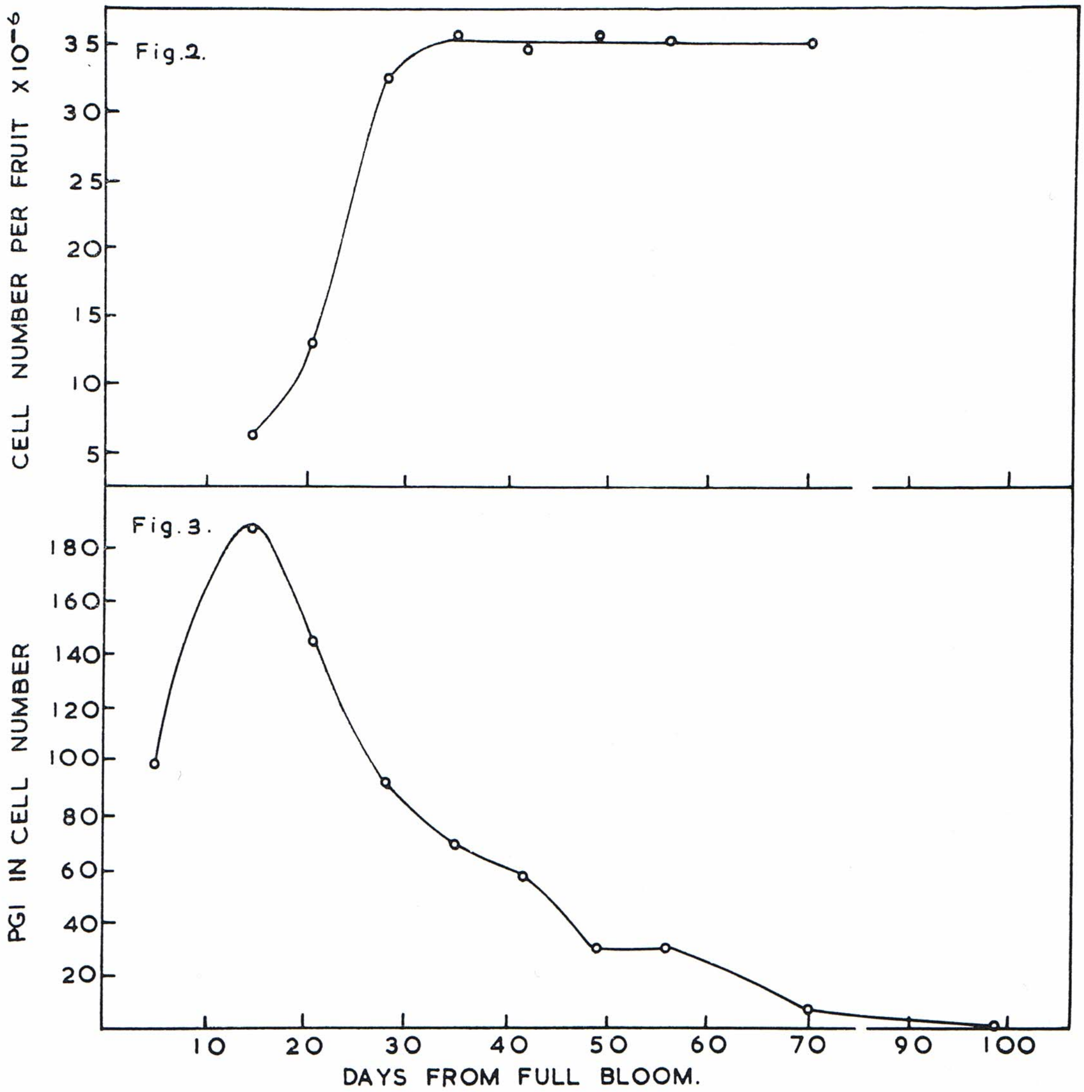

FIGURE 2

Cell number of plum fruitlets at different stages of development.

FIGURE 3

Percentage growth increment (PGI) in carrot explant cell number induced by AP fractions derived from plum fruitlets at different stages of development. The concentration of all fractions was the equivalent of 0.4 g. of tissue per 100 ml. of medium.

Purification of factors in fraction A. — To an aqueous solution of fraction A at pH 3.3 and 1° C., silver nitrate solution was added. The precipitate which separated was centrifuged down, washed with dilute silver nitrate and extracted with 0.2 *N* HCl. The resulting extract was evaporated rapidly *in vacuo* at 45° C. yielding a fraction termed C. When this was chromatographed on paper in *n*-butanol-acetic acid-water (12 : 3 : 5 by volume, solvent 1), strong activity was located at about R_F 0.70, but the active component could not be detected by chemical methods. Slight activity was detected in some bio-assays at about R_F 0.40 apparently associated with a UV-absorbing component located by UV photography [8].

In order to obtain sufficient of the factor of R_F 0.70 for characterization, the extraction procedure required considerable scaling up. To prepare larger amounts of fraction C, a simplified fractionation procedure was adopted. Plum fruitlets (30 kg.) were extracted with ethanol and the extract (dry matter 1.92 kg., optimum activity at 650 mg./l.) passed directly through 'Zeo-Karb' 225 (H) columns which were washed with 70 per cent ethanol and water before being eluted with 1.5 N ammonia. The effluent ammonia (32 l.) was evaporated *in vacuo* at 40° C. yielding a residue (102 g.) very active at 40 mg./l. The activity was precipitated with silver nitrate and recovered from the precipitate by acid extraction as previously described to give 0.86 g. of fraction C very active at 0.3 mg./l. Fraction C was extracted with solvent 1. The insoluble material was discarded while the soluble fraction was subjected to partition chromatography on a cellulose column. Fractions from the column containing the very active factor of R_F 0.70 were combined and evaporated to yield 9 mg. termed fraction D. D was very active at 0.01 mg./l. Column fractions containing the UV-absorbing base of R_F 0.40 were combined. The base was isolated as a picrate which was purified by recrystallization. From the picrate, the base hydrochloride was obtained and crystallized (22 mg. from 15 kg. of plums). This was identified as adenine hydrochloride (R_F values in several solvents, UV spectra, m. p. of picrate which was not depressed by authentic adenine picrate). Adenine was found to account for the slight activity observed at R_F 0.40.

The active factor of fraction D : Component A_1. — Fraction D was subjected to two-dimensional paper chromatography using solvent 1 and *iso*-propanol-water (4 : 1 by volume, solvent 2 ; chromatography carried out in atmosphere containing ammonia). Fraction equivalent to 1 kg. of plums was applied to each sheet. Activity coincided exactly with a component, termed A_1, detected by UV photography. A_1 was subjected to paper chromatography in seven other chromatographic systems. On all chromatograms, activity was intimately associated with A_1 which was concluded to be the active factor in fraction D.

A_1 interacted synergistically with IAA to induce cell division. In this respect it resembles kinetin and can be regarded as a kinin. In the presence of IAA and fraction B (replaceable by *myo*-inositol), A_1 increased the cell number of explants about 6 times. The exact concentration of A_1 in the media was unknown but would be less than 0.01 mg./l. The ultraviolet absorption spectra of A_1 (Fig. 4) distinguished it from the bases found in nucleic acids including the methylated purines. A_1 could be located on chromatograms by a chemical method [11] for detecting purines. Pyrimidine bases do not react in this test.

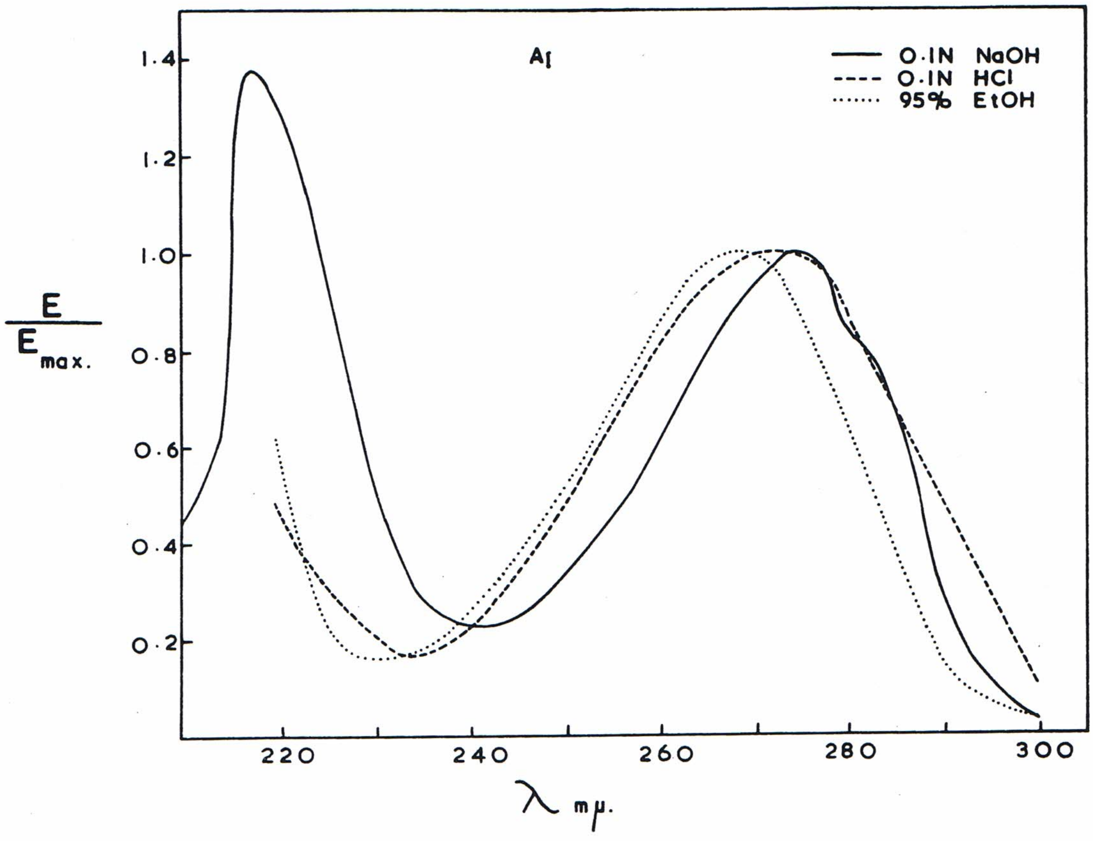

FIGURE 4
Ultraviolet absorption spectra of factor A_1 in 0.1 *N* HCl, 0.1 *N* NaOH, and 95 per cent ethanol.

Purification and Properties of a Kinin in Sweet Corn Extracts

The low level at which A_1 occurred in plum fruitlets, which are of limited availability, made isolation from this source impracticable. An alternative tissue which might contain A_1 was, therefore, sought. Immature maize caryopses (seeds) are known to be a rich source of cell-division-promoting activity [15]. Some of the activity in maize extract is retained by polystyrene sulphonic acid resins [1] and one factor eluted from this type of resin has been partially purified by MILLER [9] using methods different from those described above for the purification of factor A_1. The spectral characteristics of A_1 are similar to those of this maize factor. It appeared that maize might, therefore, also contain A_1.

The procedure used for the purification of A_1 was applied to the immature seed of a sweet corn variety of *Zea mays*. The fraction corresponding to D was subjected to two-dimensional paper chromatography. A UV-absorbing component was detected which was chromatographically and spectroscopically indistinguishable from A_1.

Activity was associated with this component which was termed M. The level at which M occurred in corn extract was about five times the level at which A_1 occurred in plum fruitlet extract.

Isolation of component M. — The UV-absorption spectra of M were very similar to those of certain N_6-substituted adenines. M might, therefore, be an adenine derivative of this type. In alkaline solution 1-methyladenine is converted into 6-methylaminopurine [2]. Other 1-substituted adenines would also be expected to undergo this rearrangement. In the purification procedure used above, 1.5 *N* ammonia was used for elution of the 'Zeo-Karb' 225 and this was evaporated at 40° C. The factor might, therefore, have been changed from 1- to an N_6-substituted- adenine. A modified purification procedure was devised which did not involve the use of alkaline solutions. In the final fraction, a component was detected which was chromatographically and spectroscopically indistinguishable from M. M, therefore, had not arisen from a 1-substituted adenine due to the use of alkaline solutions. The modified purification procedure gave a better yield of M (based on UV-absorption of M spot on chromatograms). By the procedure outlined below, M was isolated in crystalline form.

The ethanol extract of immature sweet corn seeds (70 kg.) was passed through a large column of 'Zeo-Karb' 225 (H form) which was washed with 70 per cent ethanol and then with water before being eluted with 4 *N* hydrochloric acid. The acid was evaporated *in vacuo* at 30° C. An aqueous solution of the resulting syrup was adjusted to pH 7 and extracted with *n*-butanol. To an aqueous solution (pH 3.8) of the fraction extracted by *n*-butanol, an excess of silver nitrate solution was added. The mixture was held at 1° C and the precipitate which formed was then centrifuged down, washed with dilute silver nitrate at 1° C and extracted with 0.2 *N* hydrochloric acid. The acid extracts were evaporated rapidly *in vacuo*. Water was added to the residue and evaporated to render the removal of acid more complete. 'De-Acidite' G (free base form) was added to an aqueous solution of the residue to give a pH of 2.8. The resulting solution was evaporated yielding a residue which was extracted with 95 per cent ethanol. The soluble fraction was chromatographed on cellulose using solvent 1. Fractions containing M were chromatographed on washed Whatman No. 3MM paper using solvent 2. Zones containing M were eluted with dilute formic acid which was evaporated. To an aqueous solution of the residue, saturated picric acid solution was added. The precipitate which separated was washed with water and crystallized from water to give clusters of fine needles (see Fig. 5). The yield from 70 kg. of corn was 4.2 mg. When this product was subjected to paper chromatography, only two components were detected — picric acid and substance M. The crystals were, therefore, the picrate of M. The melting point of this picrate (188-190° C) was not elevated by recrystallization.

Properties of component M. — The crystalline picrate was converted into the hydrochloride of M. Both the picrate and the hydrochloride induced cell division in carrot explants and did not appear to differ in activity. Recrystallization of the picrate did not alter its activity or the activity of the hydrochloride derived from it. The growth-promoting activity of kinetin, 1,3-diphenylurea (c. f. [12]), coconut milk and the hydrochloride of M were compared. In Fig. 6, explants (from two carrots) cultured in the presence and absence of these stimulants are shown. All media contained IAA

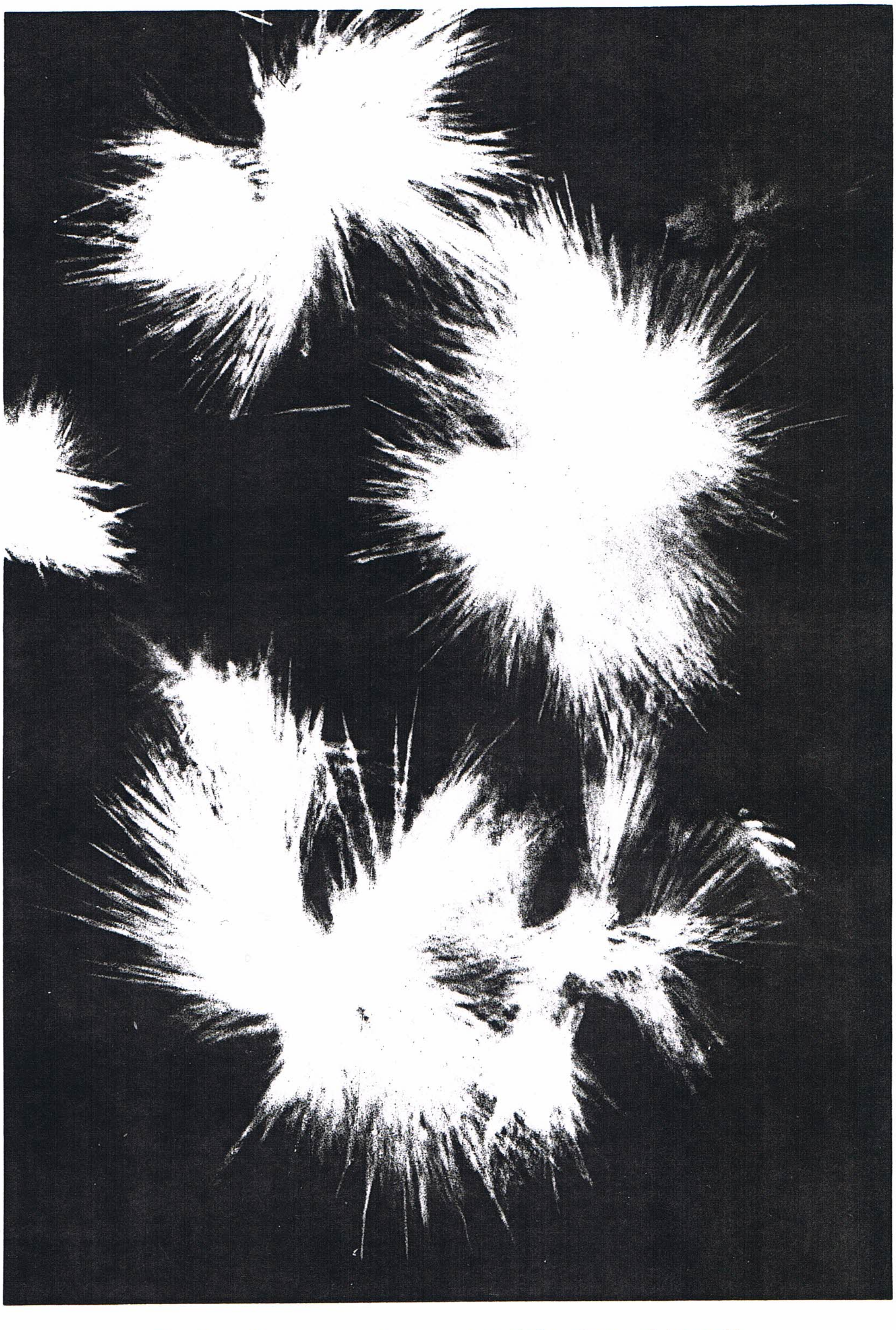

Fig. 5 — Photomicrograph of crystals of the picrate of factor M.

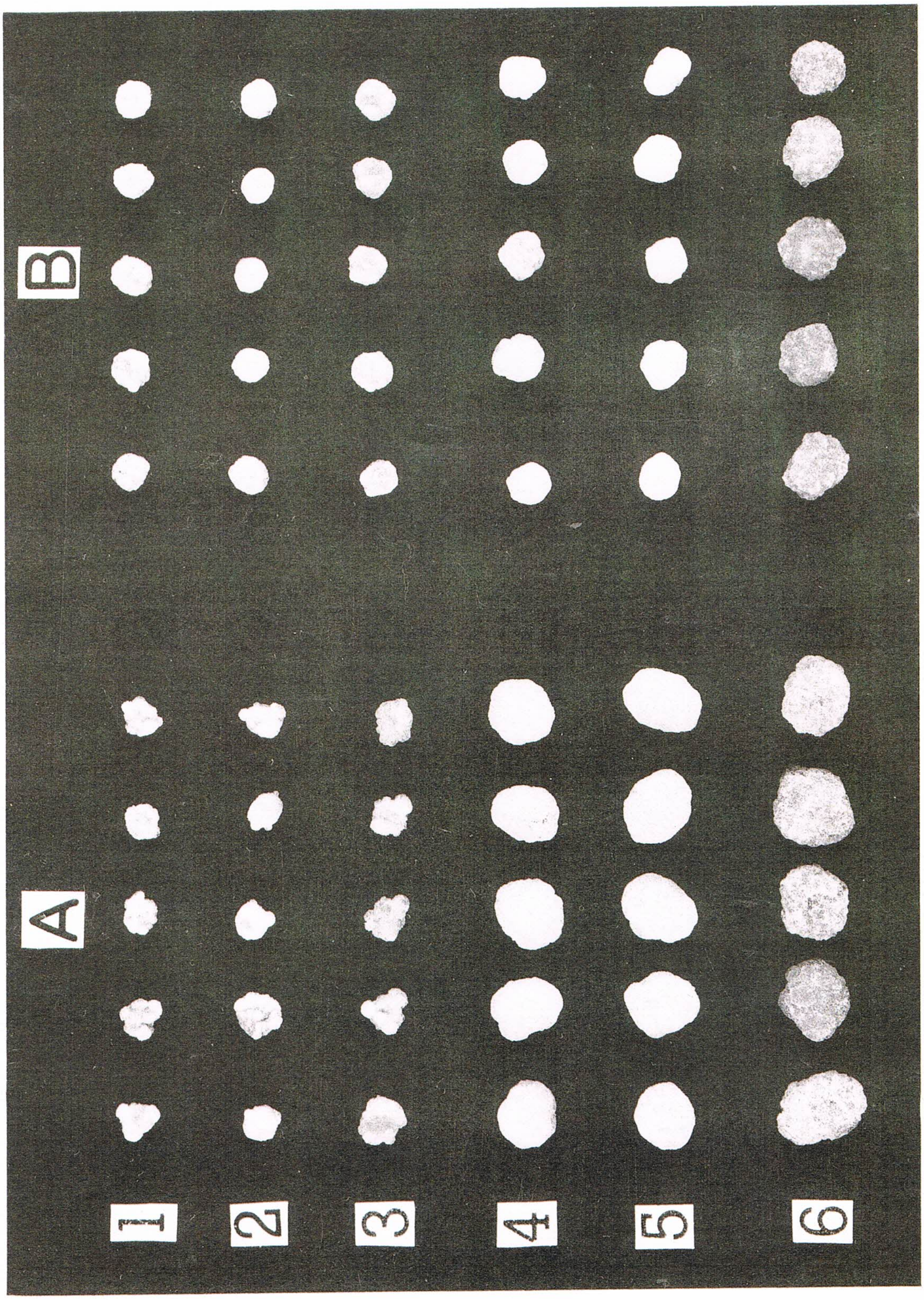

Fig. 6 — Carrot explants from two carrots (A and B) cultured on control medium (1) and on control medium containing 1,3-diphenylurea 2 mg./l. (2), kinetin 1 µg./l. (3), factor M 1 µg./l. (4), factor M 10 µg./l. (5) and coconut milk 10 per cent by volume (6). Control medium=basal + *myo*-inositol (50 mg./l.).

(2 mg./l.) and *myo*-inositol (50 mg./l.). In Table I the average cell number of these explants is recorded. 1,3-Diphenylurea (2 mg./l.) did not promote the growth of explants. At 1 μg./l. of medium, kinetin did not appreciably increase explant weight or cell number. M at this concentration induced large increments in both weight and cell number. The two carrots showed different degrees of response to M in terms of weight but not of cell number.

TABLE I

Numbers of cells in carrot explants after culture in the presence and absence of cell-division stimulants.

Medium	Average cell number per explant[1] (in thousands)	
	Carrot A	Carrot B
Control (C)[2]	104	62
C + 1,3-diphenylurea (2 mg./l.)	110	67
C + kinetin (1 μg./l.)	143	63
C + factor M (1 μg./l.)	692	390
C + factor M (10 μg./l.)	670	450

[1] Cell number determined after 21 days in culture.
[2] Control medium = basal + *myo*-inositol (50 mg./l.)

The activity of M has been confirmed with explants derived from other carrots. M at 1 μg./l. usually increased cell number about 5-6 fold; a concentration of 10 μg./l. was usually slightly more effective. Even at 0.1 μg/l., however, M showed appreciable activity. Explants cultured in the presence of M were usually light green in colour, whereas those cultured on basal medium containing *myo*-inositol remained orange-yellow.

M reacted positively on chromatograms to a spray reagent [11] considered specific for purine bases. M was oxidised by xanthine oxidase, an enzyme known to hydroxylate purine and pteridine bases. The spectral characteristics of M indicated that it was not a pteridine. M might, therefore, be a purine base. M was then degraded and the products formed were compared with known purine bases. One of the degradation products produced by mild oxidation with concentrated nitric acid was identified as adenine. M is, therefore, an adenine derivative.

M has two dissociable groups in the molecule with pK values of 4.4 and 9.8. The pK of 9.8 indicates that positions 7 and 9 in the purine ring system are unsubstituted. The dissociation at about pH 4.4 is consistent with an amino (or substituted amino) group being at position 6. If positions 1 or 3 were substituted, an imino group would occur at position 6 and a pK of about 7 would be expected. Positions 1 and 3 are,

therefore, unlikely to be substituted. Xanthine oxidase hydroxylates purines at positions 2 and 8, the introduction of a hydroxyl at both positions being accompanied by a displacement of absorption maximum to longer wavelengths by about 40 mμ. Substitution of only one hydroxyl at either position 2 or 8 produces a considerably smaller bathochromic shift. When M was oxidised by xanthine oxidase, the absorption maximum was displaced 38 mμ to longer wavelengths. M was, therefore, hydroxylated at positions 2 and 8 which it is concluded were unsubstituted.

From the above evidence it is unlikely that the purine ring system of M is substituted at positions 1, 2, 3, 7, 8 or 9. Substitution would, therefore, appear to be in the amino group on position 6, the only remaining site for substitution. This deduction that M is an N_6-substituted adenine is supported by the absorption spectra of M. These show a striking similarity to those of certain N_6-substituted adenines, *e.g.* 6-(β-hydroxyethylamino)-purine. This substance and M are almost spectroscopically indistinguishable. Further confirmation that M is an N_6-substituted adenine is provided by the absorption spectrum of the product formed when M is oxidised with xanthine oxidase. N_6-substituted adenines yield oxidation products with an absorption maximum very close to 305 mμ ; the position of this maximum appears to be very largely independent of the substituent group. M yielded an oxidation product with λ max. at 306 mμ, the absorption curve being very close to those of the oxidation products from N_6-substituted adenines.

Factor M has been named zeatin since it was first isolated in crystalline form from *Zea mays* [6]. The occurrence of zeatin in sweet corn and in plum fruitlets, two very different plant species, suggests that zeatin is a cell-division regulator of wide occurrence. Zeatin is the first substance with kinetin-like activity to be isolated in crystalline form from a plant tissue.

Literature cited

[1] Beauchesne G. (1961). Separation des substances de croissance d'extrait de maïs immature. *In :* Plant Growth Regulation. *Iowa State University Press*, Ames, p. 667-674.

[2] Brookes P. and Lawley P. D. (1960). The methylation of adenosine and adenylic acid. *J. Chem. Soc.*, p. 539.

[3] Caplin S. M. and Steward F. C. (1949). A technique for the controlled growth of excised plant tissue in liquid media under aseptic conditions. *Nature*, *163*, 920.

[4] Jablonski J. R. and Skoog F. (1954). Cell enlargement and cell division in excised tobacco pith tissue. *Physiol. Plantarum*, *7*, 16-24.

[5] Letham D. S. (1963 a). Regulators of cell division in plant tissues. I. Inhibitors and stimulants of cell division developing fruits : their properties and activity in relation to the cell division period. *New Zealand J. Botany*, in press.

[6] Letham D. S. (1963 b). Zeatin, a factor inducing cell division isolated from *Zea mays*. *Life Sciences*, in press.

[7] Letham D. S. and Bollard E. G. (1961). Stimulants of cell division in developing fruits. *Nature*, *191*, 1119.

[8] Markham R. and Smith J. D. (1949). A technique for the identification and estimation of purine and pyrimidine bases, nucleosides and related substances. *Biochem. J.*, *45*, 294-298.

[9] Miller C. O. (1961). A kinetin-like compound in maize. *Proc. Natl. Acad. Sci.* (Washington) *47*, 170-174.

[10] NITSCH J. P. (1960). Présence d'une substance type cinétine dans le jus de tomate. *Bull. Soc. Bot. France, 107*, 263-267.

[11] REGUERA R. M. and ASIMOV I. (1950). The use of silver nitrate and sodium dichromate in the detection of purines by paper partition chromatography. *J. Amer. Chem. Soc., 72*, 5781-5782.

[12] SHANTZ E. M. and STEWARD F. C. (1955). The identification of compound A from coconut milk as 1,3-diphenylurea. *J. Amer. Chem. Soc., 77*, 6351-6354.

[13] STEWARD F. C. and CAPLIN S. M. (1952). Evidence on the role of the coconut milk factor in development. *Ann. Botany* (London) *15*, 491-504.

[14] STEWARD F. C., CAPLIN S. M. and SHANTZ. E. M. (1955). Tumorous growth in relation to growth factors of the type found in coconut milk. *Ann. Botany* (London) *19*, 29.

[15] STEWARD F. C. and SHANTZ. E. M. (1959). The chemical regulation of growth (some substances and extracts which induce growth and morphogenesis). *Ann. Rev. Plant Physiol., 10*, 379.

[16] ZWAR J. A. and SKOOG F. (1963). Promotion of cell division by extracts from pea seedlings. *Aust. J. Biol. Sci., 16*, 129.
