# Supplementary material for: Zeatin: The 60th anniversary of its identification
Source: Plant Physiol. 2023 Feb 15;192(1):34–55. doi: 10.1093/plphys/kiad094 (PMC10152681; doi:10.1093/plphys/kiad094)
Supplement: kiad094_Supplementary_Data [file kiad094_supplementary_data.zip › Supplemental File S3. The Controversy ed.docx]

**Supplemental File S3. Which lab first identified zeatin? A controversy invented by Folke Skoog.** [Transcribed from notes written by Stuart Letham, 1^st^ August 2018].

Letter from Stuart Letham, 8^th^ January, 2018

“Hi Paula

Some scribbled comments on statements in Mok and Mok’s book chapter 1 [Skoog 1994] were posted on Monday. Also copy of Miller’s paper at Gif conference. This has his final statement on structure of maize factor.

I met Miller 3 times--1963 (Gif), 1967 (Ottawa), and once in Canberra in 1971. We became quite friendly and used to correspond in late 1960s and 1970s; he even offered to get me a position in Indiana Uni and thought we could work together having complementary abilities. A scan of his last letter to me is attached.

[This letter is reproduced here:

*August 24, 1994.*

*Dear Stuart,*

*I truly enjoyed hearing from you. I have wondered as to your status and activities as the years have rolled on. Like you, I am retired. Nevertheless, I have continued to do a bit of work (usually seven days each week). I continue to putter around still hoping to make some progress on the ‘leaf-to-stem-tip’ flowering signals, on some regeneration challenges, and, most of all, on the initial action of cytokinins. I wish I could tell you about some great progress, but the findings are not there, yet.*

*I do, however, continue to accumulate evidence that cytokinins – even at low levels – somehow quickly impact (inhibit) peroxidation rates of various species of tissue cultures. Well, we shall see.*

*I am removing the blank back page of the cover of the reprint – no need to pay for sending it.*

*I do hope you are well. I have had some threatening situations but am in pretty good shape now.*

*Best of regards to you*

*Carlos Miller.*]

Best regards

Stuart”

There is no suggestion of antagonism from Miller towards Letham which one would conclude would have resulted if Letham had stolen Miller’s data, as suggested by Skoog.

**The Skoog Controversy**

Letham writes: “*First what did Carlos say himself? Miller (1961) (PNAS 47: 170-174) stated his corn factor was: “*considerably purified (but not crystalised)*”. In Table 3 of Miller’s article, UV characteristics of Miller’s purest preparation are recorded. Comparison with zeatin spectra reveal considerable impurity especially at lower wavelengths. Assuming all absorbance in Miller’s spectra at λ_max_ for zeatin are due to this compound, the zeatin content in Miller’s sample can be calculated using the known constants for zeatin. On a mass basis, this gives a value of 28%, but this is of course a maximum possible amount and the correct % could be much less. This is suggested by the bioassay results. For comparable moderate responses in the soybean tissue assay, Miller’s factor requires a concentration over ten times that of zeatin. There is one certain conclusion: Miller’s factor was never purified by crystallisation and lacks purity. It may even contain inhibitory activity.*

*“In his paper at the Gif conference (1963*), [see Supplemental data File 1] *Miller again never claimed to have a pure crystalline compound, but he summarised his findings concerning the structure of his partially purified factor.*

“*UV spectra suggested an N^6^-substituted adenine. Because it reacted with KMnO_4_, he suggested it possess a double bond. Because it reacted with acetic anhydride, Miller proposed the presence of an –OH group. However, these findings can easily be explained in other ways, e.g. adjacent –OH groups, an amino group. Also there was no indication of the number of carbon atoms, C_5_, C_10_?. Shaw (Shaw 1994) assessed Miller’s data and stated that Miller and Witham were unable to propose a precise structure (there were 101 possibilities!). However, the first naturally occurring pure crystalline cytokinin (zeatin) provided the data necessary for identification (partial quote from Shaw).*

*“A passing thought: Miller never got crystals because his product was too impure (ease of crystallisation is usually related to purity.*

“*In an introduction to a 1965 paper in Plant Physiology on the partial purification of a cell-division factor from peas (Rogozinska et al.,1965),* *Skoog wrote* “Letham (13) also has used immature corn in his further work. He isolated a crystalline substance that he named Zeatin. It was more active than kinetin and appeared to be identical to the factor from plum fruitlets. Letham et al., (14) have recently characterized Zeatin as 6-(4-hydroxy-3-methylbut-2-enyl)aminopurine. The active material in Miller’s preparation is identical with this chemical (Letham, private communication)*.*”

*Again, in 1970, Skoog wrote* “Zeatin, isolated from immature corn kernels, was characterized by Letham et al. (119) as 6-(4-hydroxy-3-methyl-trans-2-butenylamino)purine. Miller earlier had isolated and partially characterized a cytokinin from the same source (137) which Letham and he (116) then showed to be identical with zeatin*”*.

“*However, two decades later, in 1994, Skoog is writing something different in the question-answer chapter [Skoog F (1994) A personal history of plant hormone and cytokinin research.**In DWS Mok, MC Mok, eds, Cytokinins: Chemistry, Activity, and Function. CRC Press, Boca Raton, FL, pp 1–14]. Referring to the Gif meeting in France, Skoog writes that* “Miller reported the isolation of a crystalline compound (from corn endosperm) that had high cytokinin activity and which he identified as an adenine derivative containing a substituent with a hydroxyl group and a double bond between the ß and ɣ carbons”.

Letham responds *“This claim by Skoog is contrary to:*

1. *Miller’s own statement in the Gif proceedings (as mentioned above).*
2. *The UV of Miller’s product that indicates appreciable impurity*
3. *The low cytokinin activity of Miller’s product relative to that of Z. Miller’s “compound” was probably too impure to crystallise. If Miller had a crystalline factor he would have quoted a melting point. None is recorded.*

Skoog wrote: “The data reported by Miller in 1961 and 1963 contradicted both Letham’s claims and common statements in textbooks that zeatin was first isolated and identified by Letham. Letham’s statement (Annu Rev Plant Physiol 1997) that Miller’s factor was “later isolated in crystalline form” may be true, but is highly misleading”.

Letham responds:

*“Clearly Z was first isolated in a pure crystalline form by Letham. This purity is evident in the MS recorded in the NZJSci 1966 (this was the MS of zeatin isolated from maize (m.p. 208-209 °C) in 1964).*

*“I never claimed to be the first person to work on isolation and identification of zeatin. However, it is reasonable to say that we were the first to obtain zeatin in a state of purity (suitable for structure determination by methods available in 1963) and to determine its exact structure.*

*“….we quote the structures and then give a reference – the authors include J.S. Shannon and I.R. McDonald. It was Shannon’s MS with only 0.6 mg that was the key. Others involved were the synthetic chemists – Shaw and Wilson in England and Cebalo in Auckland. The synthesis of Cebalo and Letham (1967) became important when Shaw’s synthesis was questioned on stereo-chemical grounds.*

Skoog continues: “Miller also isolated the compound earlier. On the basis of the available information, my conclusion is that Miller should be given priority for the isolation and composition of zeatin. The work of Letham and co-workers, together with Shaw and Wilson, established the precise location of the hydroxyl group in the side chain”.

Letham responds:

1. *Miller never isolated a pure product suitable for determination of structure. He never obtained any unequivocal structural evidence.*
2. *Skoog says we only established the precise location of the hydroxyl group in the side chain. But we also determined the structure of the side chain, that’s quite important.*
3. *Finally, we are not criticising Miller - only Skoog for ridiculous statements which climaxed in the idea that our data, involving numerous people, was stolen from Carlos.*

*Miller did make significant contributions:*

1. *He showed that sweet corn was an excellent source of cytokinin activity suitable for purification* [Note: Miller used milky kernels and Letham used near mature kernels for isolation, when in fact the greatest concentrations of cytokinins are in the immature kernels of cereals (Chen et al., 2020)].
2. *He showed that Z was a naturally occurring compound in corn when this was questioned by CSIRO people (see Miller PNAS 54, 1952 (1965). VERY IMPORTANT. “*

Skoog (1994) concluded: “This is not to detract from the valuable contributions that have since come from Letham’s lab.” These are also outlined in the review.

**Articles cited**

**Cebalo T, Letham DS** (1967) Synthesis of zeatin, a factor inducing cell division. Nature **213:**86

**Chen L, Zhao J, Song J, Jameson PE** (2020) Cytokinin dehydrogenase: a genetic target for yield improvement in wheat. Plant Biotechnol J. **18**: 614-630. doi: 10.1111/pbi.13305

**Letham DS** (1967) Chemistry and physiology of kinetin-like compounds. Annu Rev Plant Physiol **18**: 349-64

**Miller CO** (1961). A kinetin-like compound in maize. Proc Natl Acad Sci **47**: 170–174

**Miller CO** (1965). Evidence for the natural occurrence of zeatin and derivatives: compounds from maize which promote cell division. Proc Natl Acad Sci **54**: 1052-8

**Miller CO, Witham FH** (1964) A kinetin-like factor from maize and other sources. Addendum to Regulateurs de la Croissance Vegétalé. Nitsch, JP Ed., Coll. Int. Centre Nat Reserche Sci., Gif-sur-Yvette, France. [The Proceedings of the International Conference on Plant Growth Substances at Gif, France, in July 1963. Attached as Supplemental File 1]

**Rogozinska JH, Helgeson JP, Skoog F, Lipton, SH, Strong FM** (1965) Partial purification of a cell-division factor from peas. Plant Physiol **40**: 469-76

**Shannon JS, Letham DS** (1966) Regulators of cell division in plant tissues. IV. The mass spectra of cytokinins and other 6-aminopurines. NZJ Sci **9**: 833-842 [submitted before III]

**Shaw G** (1994) Chemistry of adenine compounds. *In* DWS Mok, MC Mok eds, Cytokinins: Chemistry, Activity, and Function. CRC Press, Boca Raton, FL, pp. 15-34

**Skoog F** (1994) A personal history of plant hormone and cytokinin research. *In* DWS Mok, MC Mok, eds, Cytokinins: Chemistry, Activity, and Function. CRC Press, Boca Raton, FL, pp. 1–14

**Skoog F, Armstrong DJ** (1970) Cytokinins. Annu Rev Plant Physiol **21**: 359-84
