# Supplementary material for: Zeatin: The 60th anniversary of its identification
Source: Plant Physiol. 2023 Feb 15;192(1):34–55. doi: 10.1093/plphys/kiad094 (PMC10152681; doi:10.1093/plphys/kiad094)
Supplement: kiad094_Supplementary_Data [file kiad094_supplementary_data.zip › Supplemental File S4. Roscotivine ed.docx]

**Supplemental File S4. Inhibitors of cytokinin glucosyl transferases: From zeatin and radish cotyledons to cancer treatment: the story of roscotivine**

Two enzymes extracted from radish seedlings capable of glucosylating 6-BAP were reported by Entsch and Letham (1979). Entsch et al. (1979) characterised one of these, showing it synthesized the 7-glucopyranoside of zeatin as the major product and the 9-glucopyranoside as a minor product, while 2:1 ratio of 7- and 9- glucosides of 6-BAP were formed. They proposed the trivial name cytokinin 7-glucosyltransferase. [see other Entsch et al. papers for β-(9-cytokinin)alanine synthase].

As both the 7- and 9-glucosides are inactive in most bioassays (with the exception of the 9-glucoside of BAP retarding senescence in radish leaf discs) (Letham et al. 1983b), and on the premise that inhibition of the inactivating enzyme would modify the levels of active cytokinin, a search for compounds that inhibited the natural inactivation of cytokinins was instigated. Letham synthesised two compounds, the diamino purine, 6-benzylamino-2-(2-hydroxyethylamino)-9-methylpurine and the 7-methyl analogue (Letham pers. comm), the former being identified as a potent inhibitor of cytokinin 7-glucosyl transferase (K_i_ 3.3 uM) (Parker et al., 1986). The inhibitor was active not only *in vitro* but also *in vivo*, reducing the 7-glucosylation of zeatin released from exogenous ZR in radish cotyledons, and preferentially inhibiting the formation of the 9-glucoside of BAP and elevating the levels of free 6-BAP and 6-BAP nucleotide in detached cotyledons supplied with free 6-BAP (Tao et al., 1991).

Inhibitors of cyclin-dependent kinases (cdks) were being sought due to the essential role of cdks in the regulation of the cell division cycle. Among 81 purines tested by Veselý et al. (1994), the cytokinin 7-glucosyl transferase inhibitor, 6-benzylamino-2-(2-hydroxyethylamino)-9-methylpurine (then sold by Apex Organics), was found to be one of three highly active compounds inhibiting specific cyclin-dependent kinases. It was renamed “olomoucine” and shown to provide anti-mitotic activity, likely by inhibiting certain steps of the cell cycle. When tested against human tumour lines, “olomoucine arrested cells both at the G1/S and the G2/M boundaries, consistent with the hypothesis of a prevalent effect on cdk2 and cdc2, respectively”. This led to the suggestion that “olomoucine may lead to a compound which will preferentially inhibit the proliferation of certain tumor cells” (Veselý et al., 1994). This led on to clinical trials as a cancer drug, and structural changes to make it more effective (reviewed in Zhelev et al., 2013). Olomoucine was renamed “roscovitine” (also known as R-roscovitine, CYC202 and Seliciclib) which has reached Stage 2 testing as a cancer drug. According to Nair et al. (2011) roscovitine is the first, selective, orally available inhibitor of CDKs to enter clinical trials, and is currently in phase II trials for B-cell malignancies, and lung cancer (see references in Nair et al., 2011). Seliciclib is being researched for the treatment of [non-small cell lung cancer](about:blank#Non-small_cell_lung_cancer) (NSCLC), [Cushing's disease](about:blank), [leukemia](about:blank), [HIV infection](about:blank), [Parkinson’s disease](about:blank), [herpes simplex](about:blank) infection, [cystic fibrosis](about:blank) and the mechanisms of [chronic](about:blank) [inflammation](about:blank) disorders (Wikipaedia; Holcakova et al., 2010; Zhelev et al., 2013; Shrestha et al., 2020), and as a treatment for Covid-19. A suggested rewording of the title of the review by Zhelev et al. (2013) “From Roscovitine to CYC202 to Seliciclib – from bench to bedside: discovery and development” is “F*rom zeatin and radish cotyledons to bedside*: *discovery and development of a cancer drug*.” The 81 compounds tested above included further compounds synthesized in Letham’s lab but, relative to olomoucine, these were ineffective. However, 6-benzylamino-2-(2-hydroxyethylamino)-9-methylpurine became the prototype of a group of 2,6,9-trisubstituted purines that strongly inhibit tumour cell growth.

**Entsch B, Letham DS** (1979) Enzymic glucosylation of the cytokinin 6-benzylaminopurine. Plant Sci Lett **14**: 205-212

**Entsch B, Parker CW, Letham, DS, Summons RE** (1979) Preparation and characterization using HPLC of an enzyme forming glucosides from cytokinins. Biochim Biophys Acta **570**: 124-139

**Nair BC, Vallabhaneni S, Tekmal RR, Vadlamudi RK** (2011) Roscovitine confers tumor suppressive effect on therapy-resistant breast tumor cells. Breast Cancer Res **13**: R80. doi: 10.1186/bcr2929

**Shrestha CL, Zhang S, Wisniewski B *et al.*** (2020) (R)-Roscovitine and CFTR modulators enhance killing of multi-drug resistant *Burkholderia cenocepacia* by cystic fibrosis macrophages. Sci Rep **10:**21700

**Tao GQ, Letham DS, Hocart CH, Summons RE** (1991) Inhibitors of cytokinin metabolism III. The inhibition of cytokinin *N*-glucosylation in radish cotyledons. J Plant Growth Regul **10:**179

**Veselý J, Havlicek L, Strnad M, Blow JJ, Donella-Deana A, Pinna L, Letham DS, Kato J, Detivaud L, Leclerc S, Meijer L** (1994) Inhibition of cyclin-dependent kinases by purine analogues. Eur J Biochem **224**: 771-86

**Zhelev N, Trifonov D, Wang S, Hassan M, Serafi EL, Mitev V** (2013). From Roscovitine to CYC202 to Seliciclib – from bench to bedside: discovery and development. Biodiscovery **10**: 1
